# Clinical guidelines for the prevention and treatment of osteoporosis: summary statements and recommendations from the Italian Society for Orthopaedics and Traumatology

**DOI:** 10.1007/s10195-017-0474-7

**Published:** 2017-10-20

**Authors:** Umberto Tarantino, Giovanni Iolascon, Luisella Cianferotti, Laura Masi, Gemma Marcucci, Francesca Giusti, Francesca Marini, Simone Parri, Maurizio Feola, Cecilia Rao, Eleonora Piccirilli, Emanuela Basilici Zanetti, Noemi Cittadini, Rosaria Alvaro, Antimo Moretti, Dario Calafiore, Giuseppe Toro, Francesca Gimigliano, Giuseppina Resmini, Maria Luisa Brandi

**Affiliations:** 1 Policlinico Tor Vergata Foundation, Orthopaedics and Traumatology, University of Rome Tor Vergata, Rome, Italy; 2 Department of Medical and Surgical Specialties and Dentistry, Second University of Naples, Naples, Italy; 3 Metabolic Bone Diseases Unit, Department of Surgery and Translational Medicine, University Hospital of Florence, University of Florence, Viale Pieraccini, 6, 50139 Florence, Italy; 4 Nursing Science, Center of Excellence for Culture and Nursing Research—IPASVI, University of Rome Tor Vergata, Rome, Italy; 5 Section of Orthopaedics and Traumatology, Centre for the Study of Osteoporosis and Metabolic Bone Disease, Treviglio-Caravaggio Hospital, Bergamo, Italy

**Keywords:** Fracture, Fracture liaison service, Guidance, Bisphosphonates, Denosumab, Teriparatide, Strontium ranelate

## Abstract

**Background:**

The Italian Society for Orthopaedics and Traumatology conceived this guidance—which is primarily addressed to Italian orthopedic surgeons, but should also prove useful to other bone specialists and to general practitioners—in order to improve the diagnosis, prevention, and treatment of osteoporosis and its consequences.

**Materials and methods:**

Literature reviews by a multidisciplinary team.

**Results:**

The following topics are covered: the role of instrumental, metabolic, and genetic evaluations in the diagnosis of osteoporosis; appraisal of the risk of fracture and thresholds for intervention; general strategies for the prevention and treatment of osteoporosis (primary and secondary prevention); the pharmacologic treatment of osteoporosis; the setting and implementation of fracture liaison services for tertiary prevention. Grade A, B, and C recommendations are provided based on the main levels of evidence (1–3). Toolboxes for everyday clinical practice are provided.

**Conclusions:**

The first up-to-date Italian guidelines for the primary, secondary, and tertiary prevention of osteoporosis and osteoporotic fractures are presented.

## Scope of the guidelines

These recommendations were conceived by the Italian Society for Orthopaedics and Traumatology (Società Italiana di Ortopedia e Traumatologia, SIOT), which was founded in Rome (Italy) in 1892 to promote continuous education in the field of modern orthopedics [1]. These guidelines—which are primarily intended for orthopedic surgeons, bone specialists, and general practitioners, but should prove useful to health-care professionals in general—were written to promote improved diagnosis, prevention, and treatment of osteoporosis and its consequences.

Guidelines for primary, secondary, or tertiary prevention will be described, mainly focusing on postmenopausal osteoporosis and osteoporosis in men. Although these guidelines are not intended to cover all situations, especially in the field of secondary osteoporosis, some special but not infrequent conditions that are characterized by altered bone strength and lead to some management issues (such as patients with juvenile osteoporosis and chronic kidney disease) are addressed. The identification of subjects at high risk for fractures are highlighted, and specific thresholds for intervention are defined. The management and prevention of common or rare side effects due to antiosteoporotic treatments employed in clinical practice will be addressed. Special emphasis will be given to the establishment of secondary prevention strategies (i.e., fracture liaison services) that are usually activated by secondary or tertiary referral centers and provide a link between the initial orthopedic treatment of major osteoporotic fractures (such as hip or vertebral fractures) and the initiation of therapy to prevent further fractures.

These guidelines were drafted by a scientific committee within the SIOT according to the principles of evidence-based medicine. Thus, it mainly focuses on grade A recommendations (“good evidence to recommend the action”), as based on consistent level 1 studies, and grade B recommendations, as based on consistent level 2 or 3 studies or extrapolations from level 1 studies. Thus, data obtained from large randomized controlled trials (RCTs), meta-analyses, and large systematic reviews of the best available evidence (i.e., level 1) were primarily exploited to prepare these guidelines. Cost-effectiveness was also taken into account where possible. When there was minimal evidence on a specific subject, recommendations were made based on expert opinion regarding good practice as well as the current Italian reimbursement policy. Moreover, existing knowledge was incorporated by taking into account the recently published European guidance for the diagnosis and management of osteoporosis in postmenopausal women, along with additional position papers drafted by other European societies/national institutes, and subsequent updates reported by the International Osteoporosis Foundation website [2, 3]. At the end of each of the following sections, a tool box with grade A, B, and C recommendations—which are based on the main lines of evidence described—is provided as guidance for clinical practice.

## Definition of osteoporosis and epidemiology

Osteoporosis is a systemic skeletal disease characterized by decreased bone density and a deterioration in bone quality (microarchitectural changes), leading to compromised bone strength and an enhanced risk of fractures that are not due to significant trauma [4].

The operational definition of osteoporosis proposed by the World Health Organization (WHO) is a bone mineral density (BMD), as measured using dual-energy X-ray absorptiometry (DEXA), that is 2.5 standard deviations (SD) or more below the average value for young healthy women (i.e., T-score < − 2.5 SD) in post-menopausal women and men aged ≥ 50 years [5] (Table 1). This definition originally relied on DEXA measurements at the hip. It was subsequently extended to include lumbar spine DEXA measurements. Established or severe osteoporosis is defined as when a BMD T-score ≤ − 2.5 SD is associated with a history of fragility fracture. However, it should be noted that the abovementioned criteria provide a densitometric definition of osteoporosis that can only be employed in clinical practice after a comprehensive assessment of the differential diagnosis.

**Table 1:** World Health Organization cutoffs used in the diagnosis of osteoporosis (BMD at the hip)

Normal bone	T-score > − 1 SD
Osteopenia	T-score between − 1 and − 2.5 SD
Osteoporosis	T-score < − 2.5 SD
Established (severe) osteoporosis	T-score < − 2.5 SD + fragility fracture

Osteoporosis is one of the major noncommunicable diseases, accounting for 1.75% of the global burden in Europe [6]. The prevalence of osteoporosis and its consequences (i.e., fragility fractures) is increasing worldwide in parallel with global population aging.

Osteoporotic fractures occur when a mechanical stress applied to the bone exceeds its strength. The most frequent fracture sites are the vertebral body, the proximal femur, the proximal humerus, and the distal radius. According to the WHO, fragility fractures result from low-energy trauma due to mechanical forces equivalent to a fall from a standing height or less, which would not ordinarily cause a fracture [7]. It is now believed that skeletal fragility requires both decreased bone density and poor bone quality, defined as alterations in bone architecture, bone geometry, and the material properties of the microstructural constituents such as collagen and mineral, as well as the presence of microdamage.

The probability of low-trauma fracture increases with age in both sexes. At 45 years old, the risk of such a fracture is 47.3% for women and 23.8% in men in Western Europe [8]. In women, this risk exceeds the risk for beast cancer and is similar to the risk for coronary heart disease.

The estimate for the year 2000 was 9.0 million osteoporotic fractures worldwide (1.7 million forearm fractures, 1.4 million clinical vertebral fractures, 1.6 million hip fractures), with nearly 35% occurring in Europe [6]. Figures are expected to increase over the next few decades globally, with the number of fractures expected to double by 2040 [9].

Osteoporotic fractures lead to increased morbidity and mortality, as demonstrated by the data on disability-adjusted life years (DALYs; i.e., the number of years lost due to ill health, disability, or early death), which are employed to estimate overall disease burden [10]. Indeed, in Europe, the estimated number of DALYs lost because of osteoporosis is 2.0 million [10].

In Italy, it has been estimated that about 18.5 and 10% of women and men, respectively, suffer from osteoporosis, and it is expected that the number of osteoporotic patients will increase by 25% in the next 20 years [11]. According to data from the Italian Ministry of Health, there is an annual incidence of 410,000 fragility fractures [12]. Hip fractures are undoubtedly the most direct consequences. In Italy, more than 500,000 hip fractures occurred in the elderly population and there was a 28.5% increase in hospitalizations over a period of 6 years [13]. In hip fracture patients, the 30-day and 1-year mortality rates are 9 and 36%, respectively [14, 15]. The socioeconomic burden of hip fragility fractures in elderly individuals has increased such that it has become comparable to that of acute myocardial infarction and stroke [16].

## The main types of osteoporosis

Primary or idiopathic osteoporosis, which includes juvenile, postmenopausal, and senile osteoporosis, is the most common type of osteoporosis. Secondary osteoporosis may ensue from several diseases, such as endocrine (hypogonadism, hypocortisolism, hyperparathyroidism, acromegaly, diabetes mellitus), hematological (thalassemia, multiple myeloma), gastrointestinal (malabsorption, celiac disease), rheumatic (rheumatoid arthritis, systemic lupus erythematosus, ankylosing spondylitis, scleroderma), and kidney (renal failure, chronic tubular acidosis) disorders, or from medications such as glucocorticoids, anticoagulants, diuretics, and others [11, 17] (Table 2). The characteristics of the main forms of osteoporosis will now be briefly described.

**Table 2:** Secondary causes of osteoporosis. Reproduced (with permission) from Table 7 of the* Guidance for the diagnosis, prevention and therapy of osteoporosis in Italy* (Cianferotti and Brandi [73])

Endocrinopathies Hypogonadism Hypercortisolism Hyperparathyroidism Hyperthyroidism Hyperprolactinemia Diabetes mellitus type 1 Acromegaly GH deficiency	Collagenopathies Osteogenesis imperfecta Ehlers–Danlos syndrome Marfan syndrome Homocystinuria
Hematologic diseases Multiple myeloma Myelo- and lymphoproliferative disorders Systemic mastocytosis Thalassemia	Organ transplantation
Gastrointestinal diseases Chronic liver diseases Celiac disease Inflammatory bowel diseases Gastrectomy Lactose intolerance Intestinal malabsorption Pancreatic insufficiency	Drugs: cyclosporine, thyroid hormones in suppressive doses postmenopause, anticonvulsants, anticancer drugs (aromatase inhibitors, GnRH agonists and antagonists), methotrexate, anticoagulants, loop diuretics
Rheumatic diseases Rheumatoid arthritis Systemic lupus erythematosus Ankylosing spondylitis Psoriatic arthritis Scleroderma	Alcoholism
Kidney diseases Idiopathic hypercalciuria Renal tubular acidosis Chronic kidney disease	Smoking
Rheumatic diseases Rheumatoid arthritis Systemic lupus erythematosus Ankylosing spondylitis Psoriatic arthritis Scleroderma	Drug addiction
Kidney diseases Idiopathic hypercalciuria Renal tubular acidosis Chronic kidney disease	Immobilization
Other diseases Anorexia Cystic fibrosis Hemochromatosis Chronic obstructive pulmonary disease	Severe disability

### Juvenile osteoporosis

The term juvenile osteoporosis, or idiopathic juvenile osteoporosis (IJO), is used to indicate osteoporosis in children and adolescents, and usually does not refer to any specific type of osteoporosis in these age groups.

Bone loss may occur from infancy to adolescence because of genetic mutations resulting in a reduced amount and impaired quality of the fibrous component of bone (e.g., leading to osteogenesis imperfecta), or may be secondary to a spectrum of other conditions, such as prolonged immobilization and chronic inflammatory diseases. Moreover, the use of anticonvulsants or steroids or the presence of life-threatening conditions such as leukemia may lead to fragility fractures, particularly at the spine. If an underlying cause cannot be identified, it is defined as IJO. This condition includes a group of heritable disorders characterized by low bone density and skeletal fragility, but without the extraskeletal findings reported in osteogenesis imperfecta. Skeletal involvement in patients with IJO is the result of impaired osteoblast activity and mainly affects cancellous bone [18]. Impaired activation of Wnt–β-catenin signaling was demonstrated in autosomal dominant IJO with heterozygous mutations in WNT1 [19, 20]. Recently, a new gene mutation in PLS3, which encodes plastin-3, was found in X-linked IJO, but the pathogenic role of this protein in bone diseases must be clarified [21].

The Official Pediatric Positions of the International Society for Clinical Densitometry (ISCD) defined osteoporosis in children on the basis of a history of one or multiple vertebral fragility fractures or the presence of both a clinically significant fracture history—defined as the occurrence of at least two long bone fractures by 10 years of age or three or more fractures of long bones up to the age of 19 years—in the absence of local disease or high-energy trauma, as well as a BMD Z-score ≤ − 2.0 SD at the lumbar spine and/or the total body less head (TBLH) adjusted for age, gender, and body size [22] (grade B recommendation). The total hip and the femoral neck are not preferred measurement sites for growing children because of the inherent variability in skeletal development.

In infants and children, a diagnosis of low bone mass or BMD should be reported when the BMD Z-score is less than − 2.0 SD and there is no fracture history. However, in children aged less than 5 years, interpreting the DEXA results may not be appropriate because the impact of growth delay is not quantifiable.

### Postmenopausal osteoporosis

Postmenopausal osteoporosis is a type of primary osteoporosis where the pathogenesis is associated with estrogen depletion, which enhances the bone loss that occurs with aging. This condition is characterized by a specific skeletal disease pattern, including prevalent trabecular bone loss and perforation compared to cortical bone loss, leading to site-specific fracture risks at vertebral bodies and at the distal radius [23, 24].

The rate of bone loss after menopause is a major factor in the development of postmenopausal osteoporosis. This is often characterized by high bone turnover, which is associated with a higher risk of trabecular perforation or intracortical porosity [23]. It is difficult to predict the clinical outcome for each individual due to the variability in the rate of loss after menopause [25]. In the postmenopausal period, estrogen deficiency leads to bone loss through both bone marrow expansion and endosteal resorption, whereas periosteal apposition occurs—mainly in response to mechanical stress—to counteract reduced bone strength [26, 27]. A low serum concentration of estrogen after menopause may lead to inhibited periosteal bone formation, as suggested by the results of a previous experimental study [28]. In the absence of this compensatory mechanism, the section modulus, which reflects the ability of bone to withstand bending forces, decreases because of bone marrow expansion. Both bone quality and BMD are independent predictive factors for fragility fractures [29, 30]. However, the BMD is the best predictive factor for fracture in postmenopausal women, despite the fact that bone geometry and microarchitecture are also site-specific risk factors for osteoporotic fracture (grade A recommendation).

### Osteoporosis in men

Osteoporosis is a major public health problem, even in males. Nevertheless, male osteoporosis is still underestimated and undertreated, which has significant clinical and social consequences considering that the aging male population is growing exponentially [31]. About 20% of all hip fractures occur in men, and the incidence of vertebral fractures is about half that for women [32]. However, mortality and morbidity for major osteoporotic fractures in men are higher than those for women [33].

Primary osteoporosis in men accounts for about 40% of all cases [34]. Secondary osteoporosis ensues from several conditions (i.e., hypogonadism, alcoholism, multiple myeloma, hyperparathyroidism, malabsorption, and use of corticosteroids), and is the most common type of male osteoporosis [35]. Special consideration should be given to osteoporosis associated with androgen deprivation therapy for prostate cancer, a common disease in men, because such treatment is accompanied by significant bone loss and an increased risk of fragility fractures [36]. Therefore, the exclusion of underlying pathological conditions in male osteoporosis is mandatory (grade B recommendation).

The management strategies for this condition are based on data derived from clinical trials performed on osteoporotic women [37]. This approach is simplistic, however, because the pathogenic mechanisms are substantially different in men and women, even though the definition of osteoporosis is the same for both genders.

It should be noted that only 21% of all nonvertebral fractures and 39% of all hip fractures occur in men with a T-score < − 2.5 SD. This contrasts with data obtained for the female population, in which about 64% of all hip fragility fractures occur in the osteoporotic range [38].

According to the ISCD Positions [39], bone densitometry is required to confirm a diagnosis of osteoporosis in men over 70 years or those with a history of fragility fractures (grade C recommendation). Moreover, BMD measurement using DEXA is justified for male subjects at any age in the presence of a risk factor for low bone mass, such as low body weight, high-risk medication use, or a disease or condition associated with bone loss. The WHO criteria for diagnosing male osteoporosis in individuals aged 50 years or more are currently the same as those used for women (grade A recommendation).

A recent study has also shown that biochemical tests prescribed to assess and achieve a differential diagnosis of metabolic bone diseases are not useful for identifying secondary causes of osteoporosis in older men [40]. On the other hand, Harvey et al. [41] demonstrated that algorithms of fracture risk, such as FRAX, are able to predict incident falls in elderly men.

In men, as well as in women, the most viable approach for the diagnosis of osteoporosis includes clinical assessment, the use of algorithms of fracture risk, and DEXA scans (see the section “Diagnosis of osteoporosis”).

### Secondary osteoporosis

Secondary osteoporosis is an umbrella term for all clinical conditions where bone involvement is not the main pathological finding; rather, they are characterized (at least in part) by adverse consequences of the primary disease itself or resulting from related treatments, particularly glucocorticoid (GC) use.

Bone remodeling and bone density are negatively affected by several diseases and treatments that are often associated with an increased risk of fall. Pathogenetic mechanisms of secondary osteoporosis are independent of estrogen deficiency. In fact, about two-thirds of men, > 50% of premenopausal women, but also 20% of postmenopausal women have secondary osteoporosis [42].

Secondary osteoporosis is caused by readily identifiable conditions such as malignancy, endocrinopathies, systemic inflammatory diseases, the use of certain medications (e.g., GCs, aromatase inhibitors), as well as by other diseases that are more difficult to diagnose, such as hypovitaminosis D, hyperparathyroidism, or idiopathic hypercalciuria. Young individuals, premenopausal women, men under 65 years of age, all patients with accelerated bone loss, patients with severe osteoporosis, and patients receiving antiosteoporotic treatment who experience bone loss should be investigated for other underlying causes of osteoporosis (grade B recommendation). Biochemical evaluation has a sensitivity of 92% for the diagnosis of secondary causes of osteoporosis [43]. Therefore, laboratory assessment should be prescribed to investigate the main cause of bone loss, such as hyperthyroidism, hypercortisolism, multiple myeloma, or celiac disease. It is advisable to perform a double tetracycline labeling transiliac bone biopsy to evaluate bone marrow disorders (e.g., nonsecretory multiple myeloma or mastocytosis) or defective mineralization in patients with fragility fractures and normal bone density, which are highly suggestive of secondary osteoporosis (grade B recommendation).

In systemic inflammatory disorders such as rheumatoid arthritis and inflammatory bowel diseases, chronic therapy with the GCs used to control the disease decreases osteoblast proliferation and activity and reduces osteoprotegerin (OPG) expression, which is already impaired because of the underlying disease [44]. Therefore, GC therapy for inflammatory conditions increases the bone loss, enhancing its detrimental effects on bone health. Chronic obstructive pulmonary disease (COPD) is characterized by increased production of proinflammatory cytokines, particularly TNF-α, which is associated with disease severity and loss of bone mass [45]. In this condition, patients treated with systemic GCs show a high prevalence of vertebral fracture [46]. However, this finding has not been confirmed for inhaled GCs, as demonstrated by the results of a large case–control study suggesting that the fracture risk is increased because of the disease severity in COPD rather than because of the inhaled GCs [47].

Growing evidence has demonstrated that the pathogenesis of bone fragility in diabetes mellitus of both types (1 and type 2) is multifactorial [48]. In diabetic patients, osteoporosis is characterized by low bone turnover due to decreased bone formation [49].

**Fig. 1:**
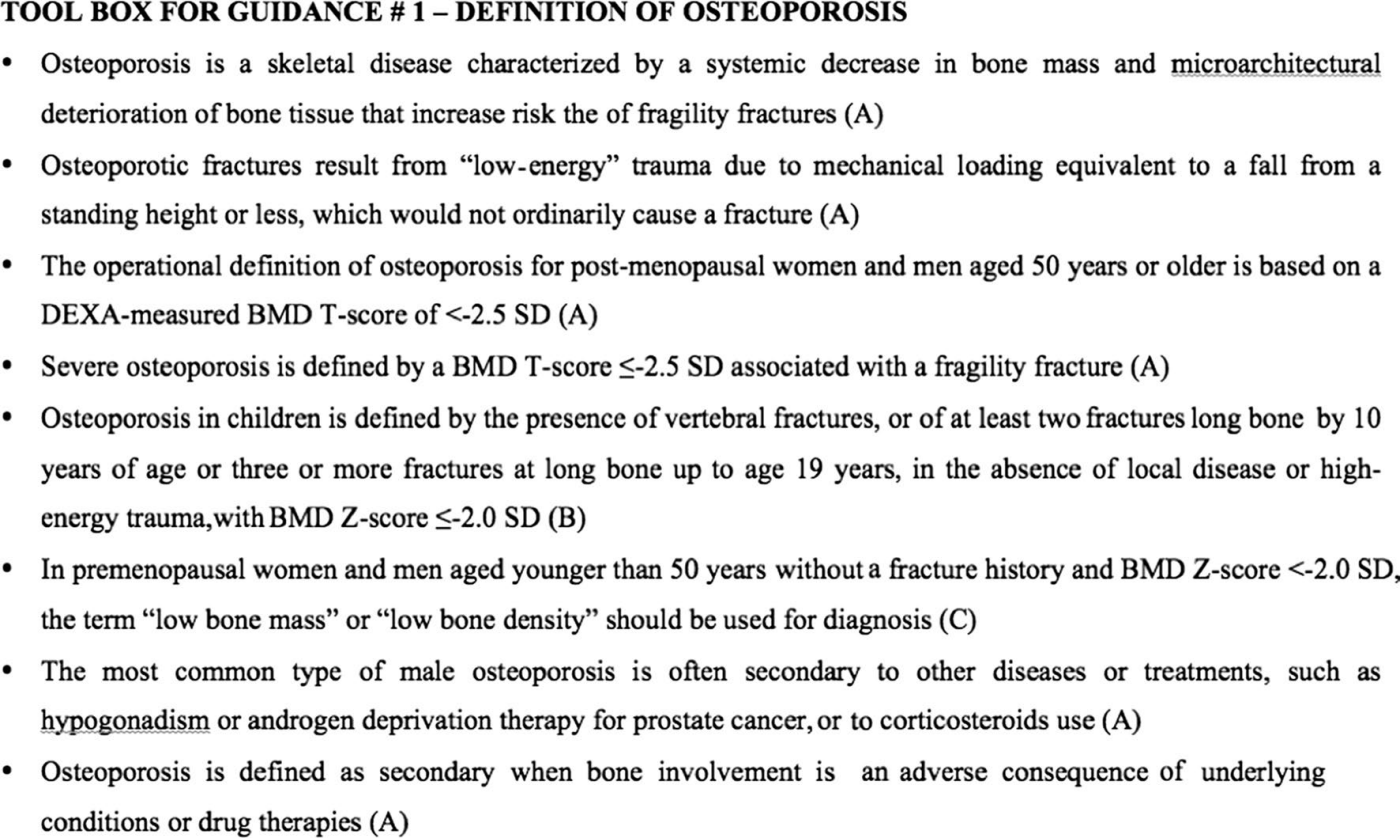
Toolbox for guidance: definition of osteoporosis

Figure 1 summarizes the recommendations for the definition of osteoporosis in a toolbox.

## Diagnosis of osteoporosis

The diagnosis of osteoporosis is based on patient medical history, careful physical examination, conventional X-rays of the thoracic and lumbar spine, bone mineral density (BMD) measurements, and laboratory investigations.

Knowledge of the medical history is essential to achieve an accurate diagnosis as well as to estimate the fracture risk. The anamnestic investigation should aim to determine the presence of any risk factor: a family history of osteoporosis and/or fragility fractures, previous fractures, nutritional habits and lifestyle, the use of medications that affect bone metabolism, the level of physical activity, and—only in women—the duration of ovarian estrogen production (grade A recommendation).

Physical examination includes assessment of patient posture, which looks for increased kyphosis of the thoracic spine, a protruded abdomen, and a loss of body height, which may be ascribed to the presence of one or more vertebral deformities (grade A recommendation).

Conventional X-ray of the thoracic and lumbar spine is useful for detecting prevalent vertebral fractures. BMD measurements are important as they can be used to better estimate the individual fracture risk, differentiate between mild and severe forms of bone loss, and select the appropriate treatment follow-up (grade A recommendation).

Laboratory tests are mandatory to exclude the main forms of secondary osteoporosis and for mineral metabolism assessment (grade A recommendation). Biochemical markers of bone turnover and vitamin D status may provide additional information on individual fracture risk (grade B recommendation). In the absence of major trauma, any fracture in adults may suggest a diagnosis of osteoporosis, so proper clinical and imaging assessment should be undertaken (grade A recommendation).

### Instrumental evaluation

The instrumental diagnosis of osteoporosis routinely includes conventional X-ray of the thoracic and lumbar spine for the detection of osteoporotic vertebral fractures, dual-energy X-ray absorptiometry (DEXA), and quantitative computed tomography (QCT) [50]. Bone quantitative ultrasonography (QUS) measures other parameters of the bone (i.e., elasticity and stiffness) that appear to be related to mechanical strength [51].

#### Conventional radiology

Identifying prevalent vertebral fractures requires a dorsal and lumbar spine assessment with X-ray or DEXA (grade A recommendation). It is important to emphasize that these fractures are often asymptomatic when they first occur and may remain undiagnosed for many years or be revealed by an X-ray examination performed for other reasons. Indeed, the presence of one or more prevalent vertebral fractures and/or other previous fragility fractures increases the relative risk of additional fragility fractures in the following year. As the number and severity of pre-existing/prevalent fractures increase, so does the relative risk for further fragility fractures [52]. It is important to exclude vertebral deformities due to congenital or acquired causes that may simulate a fragility fracture.

A morphometric analysis is required in order to quantify abnormal variations in vertebral shape. A semiquantitative method (SQ) that measures the anterior, middle, or posterior heights of the dorsal and lumbar vertebral bodies in lateral projection via conventional radiography (MRX) or with DEXA (vertebral fracture assessment, VFA) is usually employed. If one of these three heights decreases by more than 20%, the fracture is morphometrically documented [53].

Vertebral morphometry is recommended whenever there are the following red flags (grade A recommendation):

1. Acute back pain that worsens while standing and/or does not improve for several days in a person at high risk for a fragility fracture.
2. Unexplained chronic back pain in a patient with a history of a prevalent fragility fracture.
3. A height reduction of more than 4 cm compared to the maximum height reached by the subject or > 2 cm from the last control [54, 55].

#### Dual-energy X-ray absorptiometry

The gold standard for quantitative assessment of bone mineral status in adults is DEXA, performed at the lumbar spine (L1–L4) and hip (total hip or femoral neck) (grade A recommendation). It accurately and precisely measures bone density, which is the best predictor of the risk of osteoporotic fracture. A DEXA examination can also be done at the forearm (distal third of the radius), meaning that it mostly represents cortical bone, or for the total body less head (TBLH; the preferred skeletal site, along with the lumbar spine, for measuring BMD in pediatric subjects) [39]. Total body DEXA is not recommended for BMD assessment. The parameters obtained are bone mineral content (BMC) in grams, area in cm^2^, and BMD in g/cm^2^. The presence of osteophytes, vascular calcifications, and calculi could lead to an overestimation of bone mass.

BMD measurements at the lumbar spine, femoral neck, total hip, and distal third of the radius have been demonstrated to predict fragility fractures. A meta-analysis of 11 prospective cohort studies showed that a reduction in BMD of 1SD at all sites can predict fractures with a RR of 1.5 (95% CI 1.4–1.6). In the same study, lumbar spine and hip BMD measurements were able to predict site-specific fracture with RRs of 2.3 and 2.6 for vertebral and proximal femur fractures, respectively [56].

As previously pointed out, the World Health Organization has defined osteoporosis as a BMD of 2.5 standard deviations (SD) below the mean peak bone mass of young healthy adults (Table 1) (grade A recommendation). The T-score shows the bone density compared with that of a young adult (at the age of 35 years) of the same gender. The Z-score is calculated in the same way, but the comparison is made with someone of the same age, gender, race, height, and weight.

Fractured vertebrae or those with focal thickenings should be excluded from the analysis because these alterations could reduce the accuracy of the densitometric results. To obtain a comprehensive report on the spine, it is necessary to analyze at least two lumbar vertebrae. Lumbar densitometry assessment is often inaccurate after 65 years due to the aforementioned reasons, and femoral densitometric evaluation is therefore preferable after this age (grade A recommendation). At the femur, both the neck and the total femoral BMD are assessed. The lowest T-score value among those obtained at the lumbar spine, femoral neck, and total femur is considered for the diagnosis. Measurements at the distal forearm are only done when lumbar and/or femoral assessment is impractical or inaccurate, in severely obese patients, and in patients with hyperparathyroidism.

Bone densitometry is recommended for all women over 65 years and all males over 70 years of age (grade A recommendation). A prior fragility fracture, increased bone radiolucency at conventional X-ray, or clinical risk factors for osteoporosis (medications or diseases associated with bone loss) require a bone densitometric assessment, independent of age (grade A recommendation). In Italy, access to DEXA is regulated by regional exemption policies.

The interval between two densitometric assessments depends on patient characteristics. Usually a new DEXA is not performed until at least 18–24 months have elapsed since the previous DEXA, as this allows the least significant changes to be detected [39] (grade A recommendation). The percentage change in BMD and the T-score are taken into account during follow-up. When there is limited access to DEXA, vertebral DEXA assessment is preferable to hip DEXA assessment to monitor the disease and/or treatment, since it better detects the least significant changes and is thus able to guide further therapeutic choices.

#### Quantitative computed tomography (QCT) and bone microarchitecture analysis (BMA)

Quantitative computed tomography (QCT) measures not only the BMD and BMC but also the true bone density expressed in g/cm^3^. Its main advantage is its lack of interference with osteoarthritic processes. Its main limitations are the substantially higher radiation dose delivered, its reduced accuracy, and that it is relatively expensive [39]. Peripheral QCT (p-QCT), which focuses on the peripheral segments (i.e., the forearm and tibia), allows a three-dimensional reconstruction of the trabecular bone to be obtained, providing information on bone microarchitecture. High spatial resolution peripheral QCT (HR-pQCT) is a new technique that can even display the trabecular bone microstructure [57]. Bone microarchitecture analysis (BMA) is a new high-resolution digital X-ray method in which bone texture analysis is performed by means of a fractal algorithm. Although all of these techniques provide measures of bone quality, QCT, pQCT, and BMA are performed only in highly specialized centers and are not recommended for the routine evaluation of postmenopausal osteoporosis (grade A recommendation).

#### Bone quantitative ultrasound (QUS)

Bone quantitative ultrasound (QUS) analyzes the interaction between the sound signal and the tissues, providing information on bone mechanical properties. It is helpful when predicting the risk of fracture using low frequencies (200 kHz to 1.5 MHz) to analyze hand phalanx bones or the heel [58, 59]. The parameters analyzed are the speed of propagation (speed of sound, SOS), the attenuation wave (broadband ultrasound attenuation, BUA), and the amplitude-dependent speed of sound (AD-SoS). These parameters define the elasticity/stiffness characteristics of the bone, which are in part related to its density [60]. The heel-QUS method can also calculate the stiffness index (SI) and the quantitative ultrasound index (QUI), parameters derived from the SOS and BUA, which seem to be more closely related to the bone properties. When using QUS methods, osteoporosis is defined as a T-score of less than − 2.5 at the heel and less than − 3.2 at the phalanges. QUS can be recommended for epidemiological investigations and as a first-level screening tool because of its low cost and the fact that it does not require the use of ionizing radiation. QUS is a significant predictor of osteoporotic fractures but is a weaker predictor than femoral neck BMD for hip fractures. In clinical practice, it may be helpful to integrate QUS with clinical risk factors for the assessment of fracture risk [61] (grade B recommendation).

In Italy, QUS is no longer included in the recently revised criteria for reimbursement of antiosteoporotic treatments.

### Metabolic evaluation

A biochemical assessment is also recommended for the diagnosis and management of osteoporosis and fragility fractures. Biochemical assessment is not recommended in individuals without fractures who do not have a clinical or medical history of secondary osteoporosis and have a lowest T-score > − 1.0 [39]. Before prescribing a therapy, it is always important to discriminate primary from secondary forms of osteoporosis. It is a mistake to pursue a therapy for osteoporosis without having investigated the etiology (grade A recommendation). Osteoporosis may be the only manifestation of another disease, such as multiple myeloma or other malignant diseases, osteomalacia, primary hyperparathyroidism, hyperthyroidism, kidney failure, intestinal malabsorption syndromes, idiopathic hypercalciuria, male hypogonadism, Cushing’s disease, and other disorders [62]. Furthermore, bone loss may be secondary to the use of drugs such as glucocorticoids [63], lithium [64], and anticoagulants [65].

Laboratory tests commonly included in an evaluation of a differential diagnosis of osteoporosis are classified into two groups:

1. First-level exams include:
  Blood cell count
  Erythrocyte sedimentation rate (ESR)
  Serum calcium (corrected for albumin)
  Serum phosphate
  Serum protein electrophoresis
  Serum creatinine
  Alkaline phosphatase
  Urinary calcium (in 24-h urine collection).

In asymptomatic postmenopausal women with osteoporosis, this first screening has been shown to detect more than 90% of secondary causes of bone loss [43].

2. Second-level exams include:
  Serum 25-hydroxyvitamin D
  Serum thyroid-stimulating hormone (TSH)
  Serum parathyroid hormone (PTH)
  Serum ionized calcium
  Anti-tissue transglutaminase antibodies
  Urinary free cortisol, serum cortisol after 1 mg dexamethasone suppression
  Serum testosterone and SHBG (in men)
  Free light chains
  Serum tryptase (or urine* N*-methylhistamine), ferritinemia
  Free kappa and lambda light chains
  Bone marrow aspiration and biopsy, and undecalcified iliac crest bone biopsy with double tetracycline labeling when biochemical and instrumental evaluation results are inconclusive.
  Second-level screening includes analyses/exams that can be performed in patients with an extremely high suspicion of secondary causes of osteoporosis (Table 3).

**Table 3:** Biochemical testing in osteoporosis and associated diagnoses (↑ = increased; ↓ = decreased)

Test parameter	Associated condition
Blood count	Inflammatory diseases and malignancy
Serum protein electrophoresis and free kappa and lambda light chains	Multiple myeloma
ESR	↑ Differential diagnosis of inflammatory causes of vertebral deformities
Serum calcium	↑ Primary hyperparathyroidism or other causes of hypercalcemia ↓ e.g., secondary hyperparathyroidism, malabsorption
Serum phosphorus	↑ Renal insufficiency grade IV ↑ Secondary renal hyperparathyroidism ↓ Malabsorption
Alkaline phosphatase (AP)	↑ Osteomalacia, Paget’s disease
Serum PTH	↑ Hyperparathyroidism
Serum creatinine	↓ Renal osteodystrophy
25-Hydroxyvitamin D3	↑ Vitamin D intoxication ↓ Vitamin D deficiency, osteomalacia
Urine calcium/24 h	↓ Intestinal malabsorption ↑ Urinary stones
TSH	< 0.3 mU/L endogenous or caused by l-thyroxine medication as a risk factor for fractures
Testosterone in men	Hypogonadism
Anti-tissue transglutaminase antibodies	Celiac disease
Urinary free cortisol	↑ Adrenal hypersecretion
Serum tryptase or urine* N*-methylhistamine	↑ Mastocytosis
Bone marrow aspiration and biopsy and undecalcified iliac crest bone biopsy with double tetracycline labeling	Renal failure, vitamin D-resistant osteomalacia, mastocytosis, and rare metabolic bone diseases
Bone resorption parameters	High bone turnover as a fracture risk

Bone turnover markers (BTMs) are used to quantify bone remodeling (resorption/new formation cycle). They can be used to evaluate the enzymatic activities of osteoblasts, osteoclasts, and components released from the bone matrix. The levels of the BTMs are therefore proportional to the rate of bone remodeling. During life, bone metabolism varies in speed and in the balance between resorption and formation. Bone turnover increases rapidly after menopause: all BTMs are high and the loss of bone mass is rapid. Increases in resorption markers are associated with an increased risk of fracture independent of BMD [66]. Furthermore, BTMs are widely used to monitor the antiosteoporotic response to therapies in both clinical trials and daily clinical practice. In population studies of older women who had sustained a femoral neck fracture, the serum level of C-terminal telopeptide (CTX) was found to be five times higher than normal [67]. High bone turnover can be an important risk factor for fracture as it increases the loss of bone mass, resulting in microarchitectural deterioration of bone tissue [68, 69]. Changes in BMTs should always be considered in the overall clinical judgment for a person suffering from osteoporosis [69].

Bone formation markers are preferentially measured in the serum, while bone resorption markers are measured in both serum and urine (Ur). They include:

- Bone formation markers (bone alkaline phosphatase, osteocalcin, propeptides of procollagen type I (P1NP))
- Bone resorption markers (CTX, NTX, Ur pyridinoline, Ur deoxypyridinoline, Ur CTX).

Among these markers, P1NP (a bone formation marker) and serum CTX (a bone resorption marker) are the most reliable, both at baseline evaluation and in the follow-up.

Although elevated levels of BTMs have been shown to predict rapid rates of bone loss in elderly women, these laboratory tests cannot be used to diagnose osteoporosis and/or used in the clinical routine [70] (grade B recommendation). Nonetheless, BTMs have proven useful for measuring response to drug therapy and improving patient treatment compliance [71].

### Genetic evaluation

Genetic components are known to strongly influence bone mineral density (BMD) and bone architecture and turnover, so they play an important role in determining risk of osteoporosis and fragility fractures. Human twin and family linkage studies as well as animal model studies have confirmed the importance of genetic factors in the individual variance in peak bone mass acquisition, BMD, bone geometry, and metabolism and thus the predisposition to osteoporosis and related fragility fractures. Major advances in the knowledge of genetic aspects of osteoporosis and fracture risk have been made over the last two decades, principally through the study of monogenic bone diseases, linkage analyses in osteoporotic pedigrees, association case–control and population-based studies of candidate genes (studies of single genes and, more recently, the simultaneous analysis of hundreds of genes and their polymorphic variants using next-generation sequencing (NGS) techniques), and experimental crosses in animal models [72]. Currently, over 100 different common polymorphic variants within several genes that are known to be involved in bone and mineral metabolism regulation have been tested for their association with bone mass and other determinants of bone quality and fracture risk. Unfortunately, these studies have often reported inconclusive and/or contradictory results, and they have demonstrated that each individual candidate gene exerts only a relatively modest effect on bone-tissue metabolism and osteoporosis and fracture risk. Indeed, it is now well established that osteoporosis is a multifactorial complex disorder with a pathogenesis involving the interactions and synergic effects of (1) various predisposing genetic polymorphic variants in numerous genes regulating bone and mineral metabolism, (2) reversible, highly dynamic, age-, cell-, and tissue-specific epigenetic mechanisms that regulate the expression of these genes (in response to internal and external signals and changes), (3) nonskeletal risk factors that can influence the risk of falling (i.e., muscle strength, balance, and visual acuity), (4) environmental influences, and (5) dietary and lifestyle habits [72].

Nonetheless, the identification of genetic polymorphisms or epigenetic marks to refine the probability of fracture is not currently recommended in clinical practice.

When monogenic bone diseases are suspected, it is recommended that genetic analyses should be carried out in specialized research centers. Indeed, since cases of juvenile osteoporosis have been ascribed to inactivating mutations of the type 1 collagen (*COL1A1*), ERalpha (*ERα*), aromatase (*CYP19*), and low-density lipoprotein receptor-related protein 5 (*LRP5*) genes, the sequencing of these genes can be performed. When low levels of ALP are detected during first-level screening (see the next section), the sequencing of tissue nonspecific alkaline phosphatase (*TNSALP*) is recommended to exclude/confirm hypophosphatasia. The presence of decreased platelet counts may lead to a suspicion of Gaucher disease, and proper genetic screening should be performed (acid beta glucosidase, GBA) (grade B recommendation) [72].

**Fig. 2:**
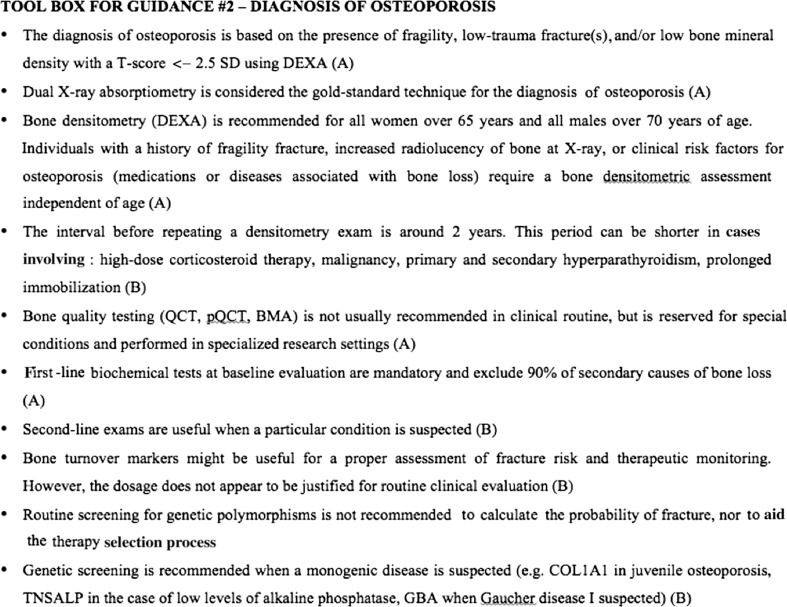
Toolbox for guidance: diagnosis of osteoporosis

Figure 2 summarizes the recommendation statements for the diagnosis of osteoporosis in a toolbox.

## Clinical risk factors and fracture risk assessment

### Clinical risk factors

The pathogenesis of osteoporosis is multifactorial, and fracture risk depends upon several independent risk factors. The overall risk of fracture in patients affected by osteoporosis derives from factors that predominantly cause a reduction in BMD and factors that are completely or partially independent of BMD, such as “bone quality” (bone geometry, microstructure, and turnover; crystalline and organic composition of the matrix) and extraskeletal factors. Many risk factors act through different mechanisms simultaneously. A low BMD, a medical history of fragility fracture, age, and a family history of osteoporosis are risk factors for osteoporotic fracture. Although BMD is used to define the diagnostic threshold, the threshold for pharmacological intervention and the absolute risk of fragility fracture depend on the independent influences of the various risk factors. It has been stated that subjects with multiple risk factors are at a higher risk of fracture than subjects with a single risk factor, including an isolated reduction in BMD. In adult osteoporosis, different factors may directly influence BMD, such as gender, calcium intake, physical activity, age of menopause, propensity to fall (such as physical disability), environmental cues, alcohol consumption, and drugs (e.g., benzodiazepines or diuretics or both) and other factors, such as age, smoking, low body weight, vitamin D deficiency. The presence of comorbidities increases fracture risk, and genetics have been shown to exert a strong influence on BMD and bone microarchitecture. Several polymorphisms (e.g., estrogen receptor, vitamin D receptor, and COLIA1) have been associated with a reduction in BMD and an increased risk of fragility bone fractures, but overall they account for only 30% of the variability in BMD and cannot therefore be taken into account when defining the risk of fragility fracture, as stated above. Table 4 lists the risk factors for osteoporosis and fragility fractures, along with their evidence levels [73].

**Table 4:** Risk factors for low BMD and fragility/low-energy fractures: levels of evidence are also shown (level 1: evidence from RCTs or metanalyses of RCTs; level 2: evidence from prospective cohort studies or poor-quality RCTs; level 3: evidence from case–control studies or retrospective cohort studies). Reproduced (with permission) from Table 1 of* Guidance for the diagnosis, prevention and therapy of osteoporosis in Italy* (Cianferotti and Brandi [73])

Risk factor	For BMD	For fracture
BMD	1	1
Age	1	1
Fragility fractures after 40 years of age	2	1
Family history of fragility fractures	2	2
Chronic corticosteroid therapy	1	1
Premature menopause (< 45 years)	1	2
Weight	1	2
Reduced calcium intake	1	1
Reduced physical activity	2	2
Smoking	2	1
Alcohol	2	3
Risk factors for falls	–	1

#### BMD

The BMD depends on peak bone mass and bone loss related to menopause and aging, and is influenced by genetic and nutritional factors, life habits, coexisting diseases, and other pharmacologic therapies. The BMD is a crucial determinant of fragility fracture risk. Many cross-sectional and prospective population studies indicate that the risk for fracture increases by a factor of 1.5–3.0 for each decrease in BMD of a standard deviation [56]. The use of bone mass measurements for prognosis depends upon the accuracy. Densitometric techniques usually have high specificity but low sensitivity, which depend on the cutoff chosen to designate high risk. However, although a reduction in BMD is an important risk factor for fragility fracture, its predictive power increases if it is evaluated together with independent factors that provide additional data complementary to the BMD.

#### Age

For both genders, fracture risk is significantly dependent on age, and fracture risk approximately doubles with each decade. Advancing age contributes to fracture risk independently of BMD. The same T-score obtained using the same technique at any one site varies in significance with age. For any BMD, fracture risk is much higher in the elderly than in the young [74, 75]. The association of age with fracture risk is probably due to a deterioration in biomechanical factors (bone architecture and bone quality), as well as the risk of multiple falls, which also increases with age.

#### Previous fractures

In both sexes, a previous fragility fracture is an important risk factor for further fractures. The most recent epidemiological studies have shown that any previous fracture, regardless of location, increases the risk of new fractures [76]. The risk also depends on the number of previous fractures. Subjects who have had three or more fractures have a roughly tenfold higher risk of new fractures than those who have not had fractures, and a two- to threefold higher risk than those who have had only a single fracture. In particular, single vertebral fractures of grade 1 according to Genant (leading to a 20–25% reduction in height) are associated with a moderately increased risk (1.5–2 times higher) of subsequent osteoporotic fractures . Two or more vertebral fractures of grade 1 or one/several fractures of grade 2 or 3 according to Genant (leading to a 20–40% and > 40% reduction in height, respectively) are very severe risk factors for further osteoporotic fractures (relative risk of between 2 and > 10). In both sexes, nonvertebral fractures after age 50 are a moderate risk factor for osteoporotic fractures, independent of BMD and age (relative risk before and after adjustment is approximately 1.9). Although a previous fracture is often related to a low BMD, the risk of new fractures is an independent risk factor.

#### Family history

A family history of fragility fractures influences fracture risk independent of BMD. A positive history of osteoporotic fractures is regarded as the most reliable prognostic indicator of a genetic risk of osteoporotic fractures. In particular, a history of femur fractures in the parents significantly increases the risk of fractures of the femur and, to a lesser degree, of all types of osteoporotic fractures.

#### Comorbidity

Several pathological disorders are associated with an increased fracture risk. In many of these conditions, the risk is mediated by the reduction in BMD. Several mechanisms are often involved, such as chronic inflammation, impairment of bone quality, the general state of health, decreased mobility, decreased muscle mass and strength (sarcopenia), and an increased risk of falls. Vitamin D deficiency is often considered an additional negative factor. The diseases usually associated with an increased fracture risk are rheumatoid arthritis, untreated hypogonadism in men and women (e.g., premature menopause, bilateral oophorectomy or orchidectomy, anorexia nervosa, chemotherapy for breast cancer, hypopituitarism, androgen deprivation therapy in men with prostate cancer), inflammatory bowel disease (e.g., Crohn’s disease and ulcerative colitis), prolonged immobility (e.g., spinal cord injury, Parkinson’s disease, stroke, muscular dystrophy, ankylosing spondylitis), organ transplantation, type 1 and type 2 diabetes, thyroid disorders (e.g., untreated hyperthyroidism, thyroid hormone suppressive therapy), and chronic obstructive pulmonary disease.

#### Medical treatments

Several drugs have been associated with an increased risk of fragility fracture. Among these, glucocorticoid therapy is the most common cause of secondary osteoporosis, mostly due to factors independent of BMD. Fragility fracture occurs in 30–50% of patients receiving long-term glucocorticoid therapy [77]. Other drugs, such as adjuvant hormone blocking therapy (aromatase inhibitors in women operated on for breast cancer, GnRH agonists in men with prostate cancer), cause a progressive reduction in BMD, but the involvement of independent risk factors is not excluded.

#### Immobility

Immobility, causing a reduction in BMD due to increased bone resorption, is a moderate risk factor for fragility fractures, with a relative risk of 1.5–2. Subjects who are limited in their mobility to such an extent that they cannot leave their home, do house work, or walk more than 100 m are regarded as immobile.

#### Smoking

Smoking is an independent moderate risk factor for vertebral fractures and peripheral fractures in both sexes, with a relative unadjusted and adjusted risk of approximately 1.2–1.8. The dependence on the number of cigarettes has not yet been adequately analyzed.

#### Risk factors for falls

Risk factors for falls play a key role in the occurrence of fractures, especially in the oldest age groups. Moreover, over 80% of nonvertebral fractures are related to falls. The main risk factors for falls are musculoskeletal and neuromuscular impairment, impaired visual acuity, hearing loss, use of psychotropic agents, diseases (e.g., Parkinson’s disease, dementia, depression, stroke-related impairment, vitamin D deficiency), use of alcohol, sedentary lifestyle, malnutrition, and environmental factors.

### Targeting risk assessment and risk charts

Despite the fact that a low BMD is still the basis for the definition of osteoporosis and the main risk factor for fragility fractures, it should not be considered alone when defining the overall risk for fracture and the single intervention threshold. The limitations of assessing bone quantity with the BMD have been discussed previously. For a given T-score, age increases the risk for fracture [75]. Moreover, while the risk for fracture varies markedly among countries, the T-score differs only minimally. Therefore, other factors may modulate fracture risk. Specific algorithms such as the Garvan calculator [78], the QFracture [79], and FRAX^®^ [80], which incorporate several risk factors in addition to age (as described above), have been developed to better define the risk for fracture and the consequent intervention threshold. Among these tools, the FRAX^®^ tool [81] has been the most extensively employed and validated in postmenopausal osteoporosis and in the other main types of osteoporosis. FRAX^®^ is a computer-based calculation tool that calculates the individual 10-year probability of major osteoporotic fractures (namely at the hip, humerus, wrist, and overt vertebral fractures) and hip fractures. It takes into account major risk factors such as age, sex, body mass index, history of fractures, previous fragility fractures, parental history of hip fractures, present tobacco smoking, previous or current long-term oral glucocorticoids, rheumatoid arthritis, secondary osteoporosis, and alcohol abuse as dichotomous variables, and includes mortality as a competing risk. Femoral neck BMD can be added, where available, to increase the sensitivity of the algorithm for predicting the risk of fractures. The calculation is available and tailored for different regions of the world as it utilizes country-specific epidemiological data on fracture and death [82, 83].

The FRAX^®^ tool does, however, have some limitations [84]. The majority of variables are discrete and not continuous, lumbar BMD is not taken into account, and there is often a discrepancy between the T-score measured at the hip and the T-score measured at the lumbar spine. Therefore, some authors have proposed adjustments to the FRAX^®^-derived risk (e.g., adjustments based on the dosage of glucocorticoids, and on the difference between the T-scores at the lumbar spine and femoral neck) [85, 86].

In the absence of a universally accepted policy in Europe for identifying individuals at high risk of fracture by population screening, a case-finding strategy is usually employed, taking into account the presence of previous or prevalent fragility fractures and any significant risk factors [2]. The presence of a previous major low-trauma fracture identifies a subject as being at high risk of (re)fracture regardless of the BMD measurement (grade A recommendation). In countries where the accessibility to DEXA measurement is high, albeit regulated by regional reimbursement policies, as in Italy, BMD assessment is useful for refining fracture risk in cases with FRAX^®^-derived intermediate risk (grade A recommendation).

Risk charts defining the 10-year probability of major osteoporotic fracture are available on the FRAX^®^ website; these are based on country-specific epidemiology data for a given BMI (for Italy, see [87].

In Italy, a FRAX^®^-derived algorithm called FRAHS was recently developed for risk assessment by general practitioners [88], based on the data from a large Italian population collected by general practitioners. Other tools, such as DeFRA (developed in Italy), have not yet been validated on a large scale.

**Fig. 3:**
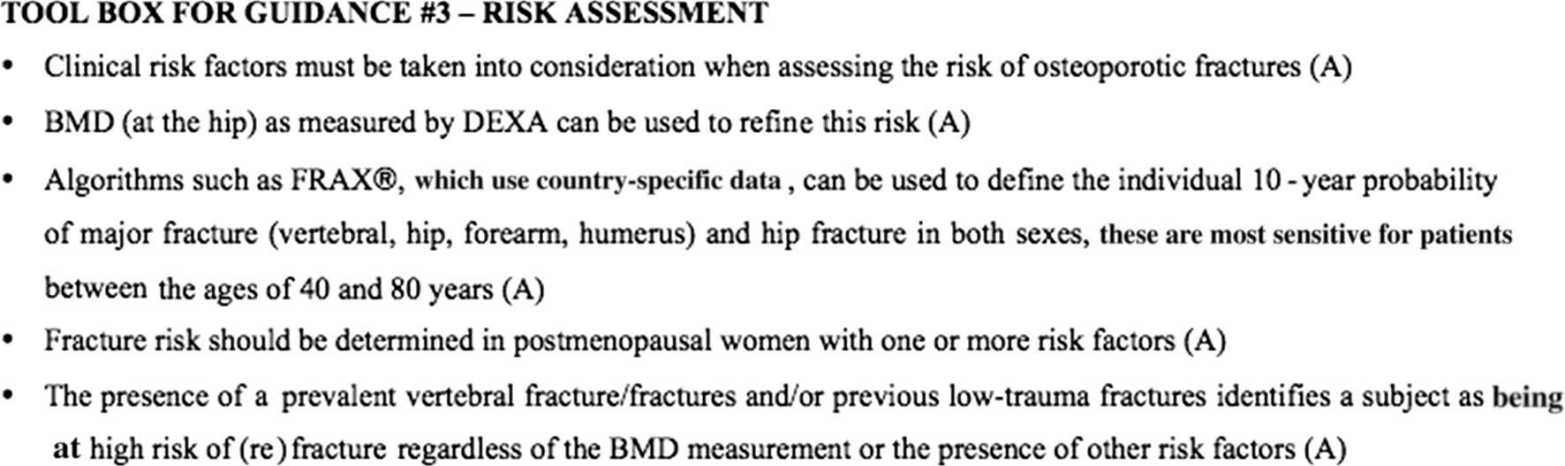
Toolbox for guidance: osteoporotic fracture risk assessment

Figure 3 summarizes the recommendation statements for osteoporotic fracture risk assessment in a toolbox.

## General strategies for the prevention and treatment of osteoporosis

### Global approaches

Health care systems should be structured to meet the needs of the patient in terms of their preferences, values, and expectations, particularly in the area of chronic disease [89, 90].

A patient-centered approach involves a partnership between health professionals and patients. For those with chronic conditions, such as osteoporosis, it means giving them an opportunity to understand their condition and the skills needed to optimize the time they invest in maintaining good health. This idea is increasingly supported by clinical evidence, particularly for chronic conditions such as diabetes and arthritis.

People with chronic diseases, including osteoporosis, require a global approach to achieve better care. The management of osteoporosis and fragility fractures, which are the most serious complications of the disease, must be multidisciplinary and comprehensive.

The basic components of the comprehensive approach are nutrition, physical activity, behavioral interventions (i.e., sun exposure, smoking habits, alcohol intake, falls screening), and/or pharmacological treatment in individuals with osteoporotic fractures or those at high risk for fractures according to the fracture liaison service strategy [91]. This approach is useful at all disease stages, from primary prevention in childhood and adolescence through subsequent ages and stages (where the aim is to achieve and maintain optimal peak bone mass and strength), right up to the tertiary prevention of elderly subjects with fragility fractures in order to counteract functional and structural regression [92] (grade A recommendation). Several studies have investigated the importance of a healthy daily life, an adequate level of physical activity, a balanced diet, and accurate screening of the risk of falls in the management of osteoporotic patients [93].

Adequate dietary intakes of calcium, vitamin D, and protein contribute to bone and muscle health and thereby reduce the risk of fragility fractures.

Physical exercise, assessment of the home for hazards (slippery floors, obstacles, insufficient lighting, handrails), assessment of visual acuity, withdrawal of psychotropic drugs, and a multidisciplinary program to reduce risk factors represent the core components of the strategy for preventing the first fall and recurrent falls. Resistance exercise performed to increase muscle strength may prevent falls, improve balance and coordination, and maintain bone strength by stimulating bone formation and decreasing bone resorption.

Another goal of the global approach to osteoporotic patients is to reduce the bone injury caused by the fall impact. There are now devices, such as padded hip protectors, that offer biomechanical protection during a fall, decreasing the force of the impact on the bone and thereby reducing the incidence of fracture of the proximal femur, particularly for high-risk institutionalized elderly individuals [94, 95] (grade B recommendation).

Pharmacologic intervention is widely used as the only approach for fragility fracture prevention in clinical practice despite nonoptimal outcomes; a comprehensive approach is considered to be the most suitable management strategy for reducing the risk of fracture.

### Exercise and fall prevention

Physical activity is any bodily movement produced by the contraction of skeletal muscle that increases energy expenditure above a basal level. Physical activity can be categorized according to mode, intensity, and purpose, and includes the following categories: occupational, leisure-time or recreational, household, self-care, and transportation or commuting activities [96]. Exercise and exercise training is defined as planned, organized, and repetitive physical activity that is frequently used to enhance or maintain physical fitness, physical performance, or specific health outcomes [97].

Several studies have investigated the timing and effect of exercise in increasing bone mass and preventing falls. The National Osteoporosis Foundation (NOF) strongly endorses lifelong physical activity at all ages, stating that proper exercise—particularly regular weight-bearing and muscle-strengthening exercises—may improve physical performance/function, bone mass, muscle strength, and balance, and can reduce the risk of falling [98].

Exercise has a positive effect on bone health, especially during the late childhood and adolescence, which are critical periods for skeletal growth and development (grade A recommendation). In a recent systematic review, Weaver et al. [99] found beneficial effects of physical activity, including dynamic resistance exercise and jumping performed at least 3 days per week, on both BMD and bone strength in youth.

In a Cochrane systematic review, Howe et al. [100] suggested that combination exercise programs, including weight-bearing activities and progressive resistance training, have a statistically significant positive effect on bone density at the spine in postmenopausal women compared to individuals that perform their usual activities. However, there is no definitive evidence supporting the benefits of exercise in women with vertebral fragility fractures [101] (grade B recommendation).

The type and amount of exercise that should be performed remain controversial. A systematic review showed that, in older adults and elderly individuals, strength exercise is effective for improving or maintaining site-specific bone mass, and multicomponent exercise programs including resistance, aerobic, high-impact, and/or weight-bearing training may help to prevent age-related bone loss, especially in postmenopausal women [102].

In a systematic review, Zehnacker et al. [103] suggested that to achieve the best results of resistance exercise in postmenopausal women, high-loading, high-intensity training for three sessions per week and for two or three sets per session is needed. Another recent systematic review showed that resistance training alone or in combination with impact-loading activities is more effective at preventing bone loss in middle-aged and older men [104] (grade B recommendation).

All of the abovedescribed systematic reviews reported that walking is not effective at preventing osteoporosis, as it only provides a modest increase in the mechanical loads applied to the skeleton. A RCT demonstrated that a specific exercise program including a combination of weight-bearing exercise with moderate/high intensity and slow progressive strength exercises could maintain and improve the hip and/or vertebral BMD as well as skeletal muscle mass and strength in postmenopausal women and in elderly people [105].

Zhao et al. suggested that resistance training was helpful for maintaining femoral neck and lumbar spine BMD in postmenopausal women. However, a subgroup analysis showed that combined protocols integrating resistance training with high-impact or weight-bearing exercises enhanced hip and spine BMD, whereas resistance-alone protocols produced only nonsignificant preventive effects on postmenopausal bone loss [106].

Zhang et al. [107] demonstrated that individuals receiving both pharmacological treatment (antiresorptive drugs) and exercise had higher lumbar spine BMD than individuals treated only with antiresorptive agents.

Physical exercise also reduces fall risk. The NICE guidelines 2013 recommend a muscle-strengthening and balance program for fall prevention [108]. Indeed, poor muscle performance and balance impairment are the key issues targeted in fall prevention programs. However, the Cochrane systematic review performed by Howe et al. [109] claimed that there is insufficient evidence to draw conclusions about the effects of exercise and physical activity programs (mixed exercise training of moderate intensity, resistance exercise, gait, balance, and functional training) on the risk of falls in older people.

On the other hand, in a Cochrane systematic review of 60 RCTs, multifactorial interventions (e.g., supervised perturbed gait exercises on a treadmill and balance training using computerized visual feedback programs) performed in hospitals significantly reduced the rate of falls, but there is no evidence for a reduced risk of falling [110].

Furthermore, a home hazard assessment and intervention, vision evaluation, and referral medication review to define the risk of falls in elderly individuals is necessary [111–113]. Additional balance intervention could be used to reduce the risk of falls. The use of whole-body vibration (WBV) could provide a significant improvement in bone loss at the lumbar spine in postmenopausal women and could be used as a complementary intervention for fall prevention [114].

Moreover, a recent RCT demonstrated that tai chi may reduce falls and injurious falls in older people more than conventional low-exercise training, and that this reduction can be maintained for at least 1 year [115].

### Nutrition

Nutrition plays a key role in the management of osteoporosis. Daily adequate calcium, vitamin D, and protein intake is the preferred option, along with good sun exposure (in summer months at a latitude > 37°N). Vitamin D insufficiency and deficiency are common among older people and have detrimental effects on bone health and neuromuscular function. Levels of serum 25(OH) vitamin D (the marker of vitamin D status) of less than 20 ng/ml are associated with mineralization defects. In subjects at high risk for fractures, a target of 30 ng/ml should be recommended [116] (grade A recommendation).

The use of combined calcium and vitamin D3 supplementation has been proven to reduce fracture rates in institutionalized older people. Although there is emerging evidence that correction of hypovitaminosis D may reduce propensity for falling [111], its relative contribution to fall risk reduction and the appropriate dosing regimen are uncertain [117].

Supplementation of calcium plus vitamin D was significantly related to total and hip fracture risk reduction in both community-dwelling and institutionalized middle-aged to older adults [93].

In elderly patients with severe hypovitaminosis D [25(OH) vitamin D < 10 ng/ml], the administration of cholecalciferol (vitamin D3) 50,000 IU per week for 8 weeks, or the equivalent of 6000 IU per day, followed by a maintenance regimen with 1500–2000 IU per day is recommended [118] (grade A recommendation). The use of calcifediol (25(OH) vitamin D3) is an effective alternative strategy to treat hypovitaminosis D, as demonstrated by the RCT performed by Bischoff-Ferrari et al. In that study, the oral administration of 20 µg per day (4 drops) or 140 µg weekly of calcifediol resulted in a significantly more efficient and rapid increase in the serum concentration of 25(OH)D_3_ and PTH suppression compared with cholecalciferol [119]. Given the different pharmacokinetics and smaller distribution volume, the administration of calcifediol should be preferred in conditions characterized by impaired 25-hydroxylation, obesity, and malabsorption, and when a rapid correction of vitamin D status is needed in order to begin an antifracture treatment [120] (grade B recommendation). Despite the positive safety profile of this vitamin D metabolite, the serum dosage of calcifediol and the level of urinary calcium should be monitored carefully during supplementation (grade B recommendation).

A low calcium intake, especially in young adults, has a role to play in the prognosis of osteoporosis. An increase in dietary calcium intake through the consumption of calcium-rich foods (e.g., milk, yogurt, cheese) represents the first step to correcting a negative calcium balance. The recommended intake of calcium (RNI) is at least 1000 mg daily and 800 IU of vitamin D per day in men and women over 50 years. Dairy products that are fortified with calcium and vitamin D and provide at least 40% of the RNI of calcium (400 mg) and 200 IU of vitamin D per portion are valuable options (e.g., yogurt or milk) [2, 116] (grade A recommendation). When dietary sources are not sufficient to provide daily requirements, calcium supplements can be administered [121] (grade A recommendation).

Caloric intake decreases with age, as does protein intake. It has been established that dietary proteins have a direct effect on key regulatory proteins and growth factors involved in muscle and bone growth, such as mammalian target of rapamycin (mTOR) and insulin-like growth factor-I (IGF-I). Branched-chain amino acids lead to the activation of mTOR and aromatic amino acids (which are particularly prevalent in dairy proteins), causing increased IGF-I, which results in greater muscle mass and strength. Protein intake has a positive impact on bone health at all ages [93]. A meta-analysis demonstrated a positive association between protein intake and BMD, BMC, and a reduction in bone resorption markers [122].

Moreover, combined protein supplementation and resistance exercises resulted in greater gains in muscle mass and strength. The recommended average daily intake of protein is at least 1.0–1.2 g/kg/BW, including at least 20–25 g of high-quality protein (such as protein supplied by dairy products) with each main meal (breakfast, lunch, dinner) during the day [121] (grade A recommendation). Recently, it was hypothesized that not only vitamin D but also other vitamins and minerals might play a role in maintaining bone health, although there are contrasting data in this context [123]. An inadequate intake of other micronutrients may contribute to the progressive age-related loss of muscle mass and strength in the elderly [124]. A recent scoping review provided a small amount of evidence supporting the use of micronutrients for healthy aging. In particular, beta-alanine, calcium, creatine, fluorides, leucine, magnesium, omega-3 fatty acids, potassium, vitamin B6, vitamin B9, vitamin B12, vitamin C, vitamin D, vitamin E, vitamin K2, and zinc can maintain or improve muscle strength and bone mass [125] (grade B recommendation).

### Particular approaches in high-risk groups

Patients at high risk of fragility fractures and falls include those with comorbidities such as Parkinson’s disease (PD), multiple sclerosis (MS), or neuromuscular disease (NMD), which might impair muscle and bone health [126]. A recent review investigated bone loss in patients with PD and observed a lower BMD in those patients than in age-matched controls. Both reduced bone mass and frequent falls may explain the increased fracture risk in these patients [127]. However, the efficacy of balance exercise at preventing fractures in PD patients is not supported by sufficient evidence [128]. Osteoporosis and fractures are also a major cause of morbidity in patients with MS. Early intervention can improve their bone health and decrease fracture risk. Osteoporosis should be treated with a comprehensive approach that includes lifestyle changes, increasing physical activity, optimizing serum levels of 25(OH)D_3_ and calcium intake, and the use of antiresorptive therapy. Resistance training might also be useful for increasing bone and skeletal muscle strength, improving balance, and reducing the risk of falls in patients with MS [129].

Neuromuscular diseases such as Duchenne and Becker muscular dystrophies are characterized by reduced muscle mass and strength, which can lead to significant bone loss [130]. No guidelines regarding the appropriate treatment of bone involvement in these conditions are currently available, and it would be desirable to treat these patients as well as postmenopausal women.

Individuals with a history of recent fracture should also be considered at high risk of a new incident fragility fracture. This category of osteoporotic patients has recently been denoted “individuals at an imminent risk of fracture.”

It is now recognized that the number of reported falls is more predictive of limb fractures than a low BMD [131]. Management of the risk of falls is the first step in the detection of patients at a high risk of fracture. The use of padded hip protectors may reduce the risk of fragility fracture in subjects at a high risk of falls. A recent review underlined that hip protectors, when correctly worn, can decrease hip fracture risk and both morbidity and mortality in the elderly, especially in institutionalized individuals [132]. However, a Cochrane systematic review demonstrated that there was little evidence that the use of hip protectors reduces the incidence of hip fracture in older people in institutional settings; it had little or no effect on falls and adverse events (skin irritation). Nevertheless, the current best evidence suggests that the use of hip protectors may slightly increase the risk of pelvic fracture [133] (grade B recommendation).

**Fig. 4:**
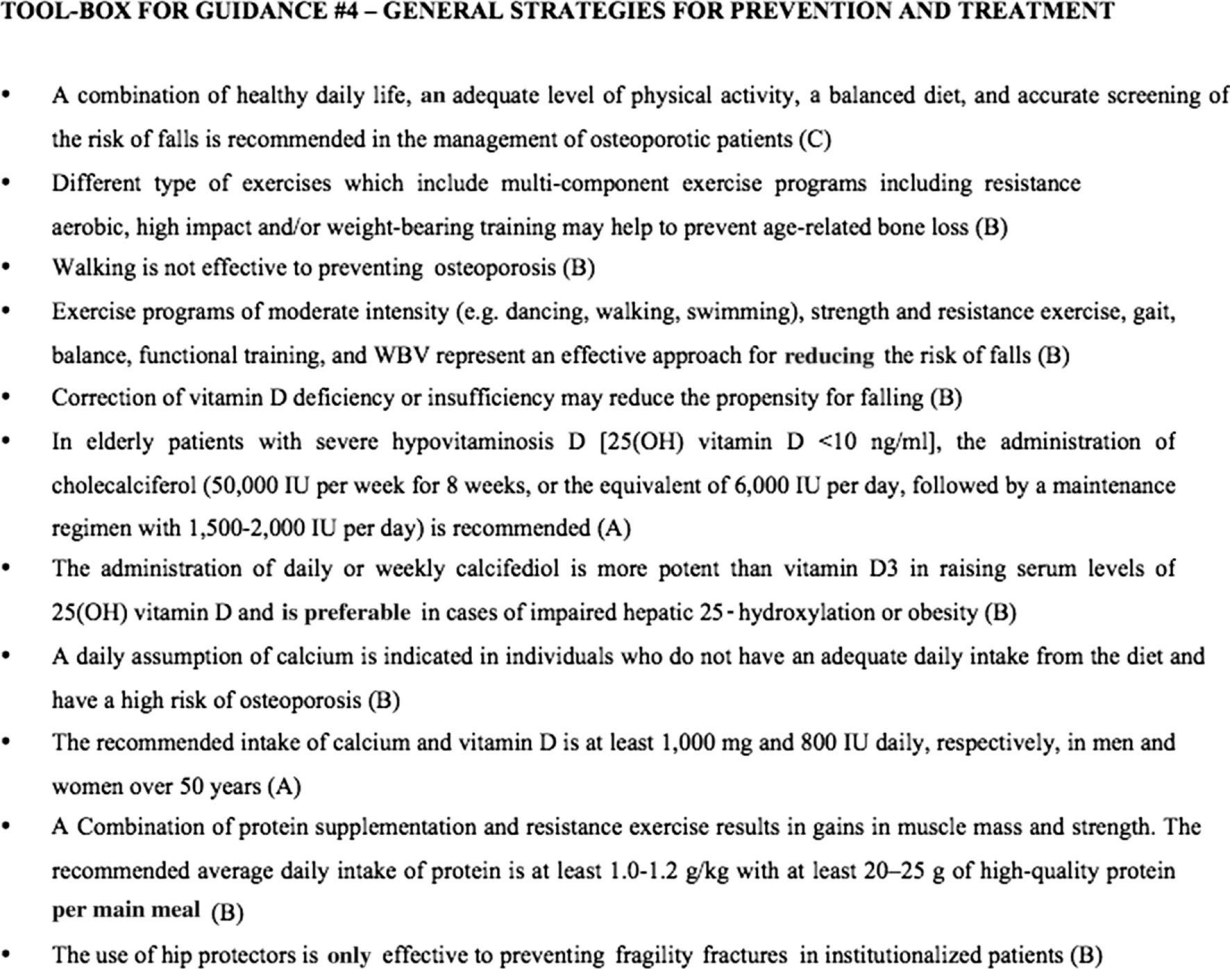
Toolbox for guidance: general strategies for prevention and treatment

Figure 4 summarizes the recommendation statements regarding general strategies for the prevention and treatment of osteoporosis as a toolbox.

## Pharmacologic treatment

Together with the general guidelines described above, pharmacologic treatment must be undertaken in order to decrease the risk of fracture in individuals at high risk.

### Age-dependent thresholds for intervention

A history of a previous major low-trauma fracture/fractures or the presence of a prevalent vertebral fracture/fractures as assessed by vertebral morphometry identifies subjects requiring treatment independent of a BMD assessment (grade A recommendation).

**Fig. 5:**
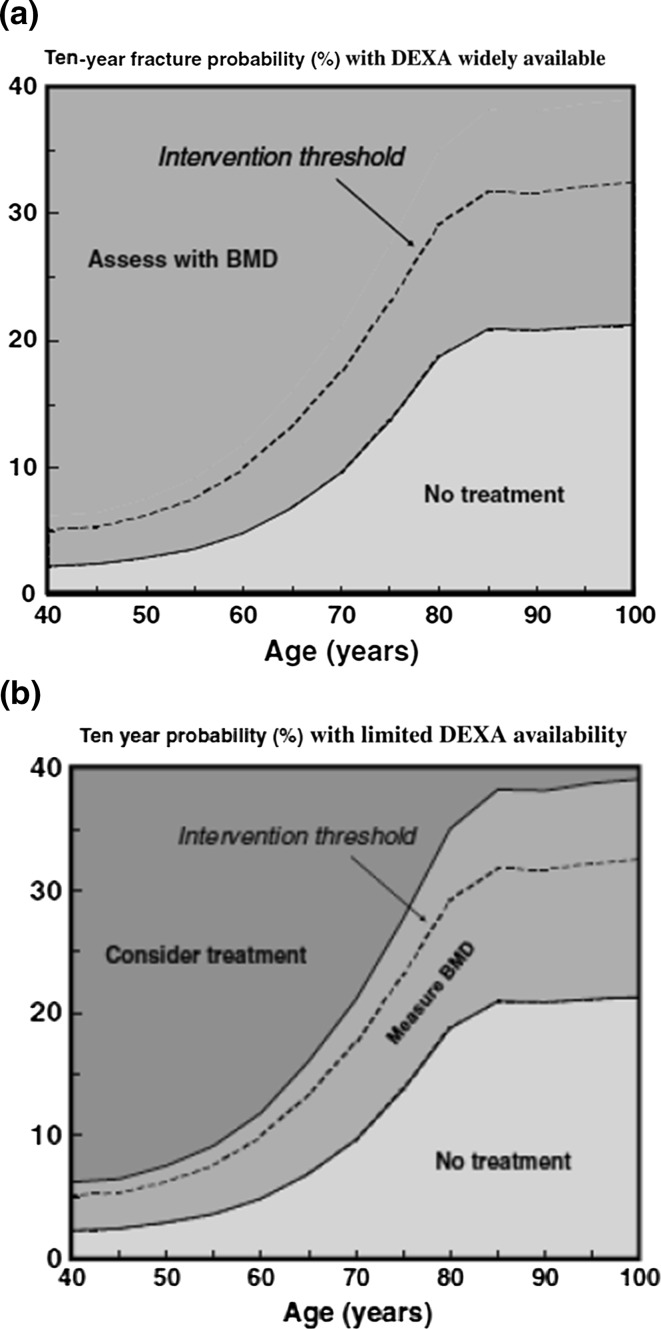
**a** Assessment of fracture risk in postmenopausal women when DEXA is widely available. **b** Assessment of fracture risk in postmenopausal women when access to DEXA is limited (reproduced from [2])

In women without prior fragility fractures, and when a BMD assessment is widely available (as it is in most areas of Italy, depending on regional exemption policies), BMD assessment by DEXA can be employed to further refine the risk for fracture. The 10-year probability of major osteoporotic fracture and the threshold for treatment in women without prior fragility fractures with one or more risk of fracture are shown in Fig. 5. According to this, treatment can be recommended for postmenopausal women when the fracture probability as calculated by FRAX exceeds the intervention threshold at a given age (grade A recommendation). In fact, the intervention threshold depends greatly on age. In older subjects it is almost equal to 20%, while it appears to be less than 5% in younger subjects [2].

In areas with only limited access to DEXA, or in the absence of criteria leading to an exemption from the fee for DEXA, FRAX calculated without BMD can be used to define the fracture probability at which to assess BMD and intervene with pharmacologic treatment. In subjects where the calculated risk lies within the intermediate area, it is advisable to assess BMD in order to better refine the 10-year probability risk [2].

### Approved drugs for postmenopausal osteoporosis and indication for treatment

Approved drugs for the treatment of postmenopausal osteoporosis include antiresorptives such as bisphosphonates, denosumab, and selective estrogen receptor modulators (SERMs), the proformative agent teriparatide, and the antiresorptive/proformative compound strontium ranelate. All these therapies have been shown to reduce the risk for vertebral fractures, while some of them also reduce the risk for nonvertebral fractures, including hip fractures (Table 5) (grade A recommendation).

**Table 5:** Approved drugs for postmenopausal osteoporosis. Reproduced (with permission) from Table 6 of the* Guidance for the diagnosis, prevention and therapy of osteoporosis in Italy* (Cianferotti and Brandi [73])

Drug	BMD	Vertebral fx	Nonvertebral fx	Hip fx
Alendronate	1	1	1	1
Clodronate 800 mg/day/os	1	1	1	
Etidronate	1	1		
Ibandronate	1	1	1^c^	
Risedronate	1	1	1	1
Zoledronate	1	1	1	1
Teriparatide	1	1	1	
PTH1-84	1	1		
Strontium ranelate	1^b^	1	1	1^c^
ERT^a^	1	1	1	1
Raloxifene	1	1		
Bazedoxifene	1	1		
Denosumab	1	1	1	1

Each number in a table cell is the level of evidence for the effect of the drug on BMD or fracture risk (fx) at a particular site

^a^No longer recommended because of side effects

^b^Also determined by strontium high-molecular weight per se

^c^As demonstrated by post hoc analyses

#### Bisphosphonates

Bisphosphonates (BPs) are analogs of inorganic pyrophosphate and inhibit bone resorption. They are able to block osteoclastic activity through a mechanism of action that depends on the presence or absence of an amino group. All bisphosphonates developed so far for the treatment of skeletal diseases are able to reduce bone turnover in a dose-dependent manner with proportional increases in bone density and to decrease fracture risk (grade A recommendation). Bisphosphonates (BPs) are poorly absorbed (0.5–5%) in the gastrointestinal tract. BPs are contraindicated in patients with hypocalcemia, gastrointestinal disease, and renal impairment (serum creatinine above 200 μmol/l or creatinine clearance below 30 ml/min), and in those who are pregnant or lactating (grade A recommendation).

The BPs that are currently registered in Europe (and in Italy) for the treatment of postmenopausal osteoporosis are:

- Etidronate
- Clodronate
- Alendronate
- Risedronate
- Ibandronate
- Zoledronate (zoledronic acid).

Etidronate and clodronate are bisphosphonates that lack an amino group. In menopausal women, these drugs increase spine BMD and maintain a stable femoral neck BMD. The recommended dose of etidronate is suboptimal in order to avoid negative effects on bone mineralization. Clodronate has proven to be effective at reducing clinical fractures at a dose of 800 mg/day per os. Parenteral administration is also possible for clodronate (100 or 200 mg/every 15 days intramuscularly), but a similar efficacy at reducing fractures of the i.m. form has not been demonstrated by comparative studies [134]. For these reasons, etidronate and clodronate are second-choice drugs for the treatment of osteoporosis.

For alendronate and risedronate, there is documentation of broad-ranging efficacy in the prevention of vertebral and nonvertebral fractures (including hip fractures), with a reduction in fractures of about 40–50% in 3 years. The antifracture effectiveness of these two drugs has been demonstrated upon daily administration.

In postmenopausal women with osteoporosis, alendronate 10 mg daily has been shown to reduce vertebral, nonvertebral, and hip fractures. Approval for a 70 mg once-weekly formulation of alendronate was granted on the basis of a bone mineral density bridging study [135]. Recently, alendronate became available as an oral solution to be administered once weekly (70 mg) in order to decrease side effects and maximize absorption. Recent data indicate that the use of proton pump inhibitors in combination with oral bisphosphonates may reduce antifracture effectiveness.

Risedronate 5 mg daily or 35 mg once weekly by mouth is also approved for the treatment of postmenopausal osteoporosis (to reduce the risk of vertebral fracture) as well as for the treatment of established postmenopausal osteoporosis (to reduce the risk of hip fracture). In a large population of elderly women, risedronate significantly decreased the risk of hip fracture, an effect that was greater in osteoporotic women. Approval for the 35 mg once-weekly formulation and for the 75 mg × 2 monthly formulation (administered each day for two consecutive days) was granted on the basis of a BMD bridging study [136].

Ibandronate was approved on the basis of studies using a dose of 2.5 mg/day. At this dosage, the drug is effective only at reducing the risk of vertebral fractures. In a post hoc analysis of high-risk women (femoral neck BMD T-score below − 3.0 SD), a significant reduction in nonvertebral fractures was shown. Ibandronate was subsequently marketed at a dose of 150 mg/month (oral administration) or 3 mg i.v./3 months. These dosage regimens are approved for the treatment of osteoporosis in postmenopausal women at an increased risk of fracture [137, 138].

Oral BPs (i.e., alendronate, risedronate, ibandronate) should be used with caution in patients with upper gastrointestinal disease because of possible side effects. Side effects of oral BPs include upper gastrointestinal symptoms and bowel disturbance. Alendronate should be taken after an overnight fast and at least 30 min before the first food or drink (other than water) of the day or any other oral medicinal products or supplementation (including calcium). Tablets should be swallowed whole with a glass of plain water (~200 ml) while the patient is sitting or standing in an upright position. Patients should not lie down for 30 min after taking the tablet.

Zoledronate (zoledronic acid) (5 mg/i.v./year) was registered for treatment on the basis of a study that clearly documented an effect on the risk of vertebral, nonvertebral, and hip fracture after 3 years of treatment. A study of an extension of the treatment to 9 years showed that the bone mass values at the femoral level remained stable. Nevertheless, there were no significant differences in BMD, bone turnover markers, and new fracture incidence from the group that discontinued treatment 6 years previously. The drug has proven itself able to reduce the risk of new clinical fractures and mortality when administered 2 weeks after a hip fracture [139]. Side effects of zoledronic acid include an acute phase reaction (see above), usually only after the first infusion, and gastrointestinal symptoms. An increase in atrial fibrillation, reported as a serious adverse event, was also seen in the main phase III trial, although this finding has not been replicated in other trials involving zoledronic acid. Zoledronic acid is given as an intravenous infusion over a minimum period of 15 min.

Because of concerns over possible adverse effects of long-term bisphosphonate therapy (i.e., osteonecrosis of the jaw (ONJ) and atypical fractures), the need to continue treatment should be reviewed at regular intervals.

Based on the available data, it is recommended that the risk should be reassessed after 5 years for alendronate, risedronate, or ibandronate and after 3 years for zoledronic acid. In patients at a high risk of fracture, a continuation of treatment without the need for further assessment can generally be recommended (grade A recommendation).

Withdrawal of treatment from alendronate, ibandronate, or risedronate is associated with decreases in BMD and increased bone turnover after 2–3 years for alendronate and 1–2 years for ibandronate and risedronate. When treatment is discontinued after 3 years of zoledronic acid therapy, the beneficial effects on BMD continue for at least another 3 years. For most treated individuals, the treatment should be stopped after 3 years, and the case for continuation of therapy reviewed 3 years later. Individuals with a previous vertebral fracture or a pretreatment hip BMD T-score ≤ − 2.5 SD may be at increased risk of vertebral fracture if treatment is stopped [140].

#### Denosumab

Denosumab is a humanized monoclonal antibody capable of neutralizing RANKL, a cytokine that interacts with the RANK receptor on the membrane of pre-osteoclasts and mature osteoclasts. In this way, it affects osteoclast recruitment, maturation, and survival. Subcutaneous administration is followed by a reduction in osteoclastic bone resorption and, subsequently, a reduction in neoformative activity; for this reason, it is an antiresorbitive drug, like bisphosphonates. The most significant differences from BPs are (a) the effect, which ceases immediately upon the disappearance of the drug from circulation (therefore, if treatment is discontinued and the patient is still at a high risk of fracture, a rapid re-evaluation to consider whether to start an alternative treatment is recommended), (b) its uniform action on all skeletal structures irrespective of bone turnover, which results in greater pharmacological activity in the cortical bone, and (c) that chronic therapy is associated with a continuous densitometric increase, in contrast to what happens with other antiresorptive drugs, which plateau in BMD after 3–4 years of therapy, particularly at the cortical level.

Denosumab is approved for the treatment of osteoporosis in postmenopausal women at increased risk of fracture, and is given as a subcutaneous injection of 60 mg once every 6 months. In postmenopausal women, the antifracture effectiveness has been documented for vertebrae (− 68% after 3 years of therapy), femur (− 40% after 3 years of therapy), and nonvertebral sites (− 20% after 3 years of therapy). Denosumab has also demonstrated antifracture efficacy in women with breast cancer treated with aromatase inhibitors and in men under antiandrogen treatment for prostate cancer. In the most severe forms of osteoporosis, an additional BMD gain has been documented when denosumab is combined with teriparatide in the sequence teriparatide–denosumab but not vice versa [141].

Like BPs, denosumab is contraindicated in women with hypocalcemia or hypersensitivity to any of the constituents of the formulation. Its use is not recommended in pregnancy or in the pediatric population (age ≤18 years). No dose adjustment is required in patients with renal impairment. The safety and efficacy of denosumab in patients with hepatic impairment have not been studied. Hypocalcemia should be corrected and prevented by ensuring an adequate intake of calcium and vitamin D before initiating therapy. Side effects include skin infection, predominantly cellulitis, and hypocalcemia. Hypocalcemia is an identified risk in patients treated with denosumab, and one that increases with the degree of renal impairment. Pre-existing hypocalcemia must be corrected prior to initiating therapy. An adequate intake of calcium and vitamin D is important in all patients, especially in those with severe renal impairment. Monitoring of calcium levels and an assessment of calcium intake should be conducted prior to each dose of denosumab and within 2 weeks after the initial dose in patients predisposed to hypocalcemia (e.g., patients with severe renal impairment, creatinine clearance < 30 ml/min), or if suspected symptoms of hypocalcemia occur, or if otherwise indicated (grade A recommendation). Patients should be advised to report symptoms of hypocalcemia.

#### Potential adverse events of antiresorptive therapy: osteonecrosis of the jaw (ONJ) and atypical fractures

Antiresorptive therapy for malignant diseases (bone metastases, malignant hypercalcemia, etc.) in doses ten times higher than those used for the management of osteoporosis is associated with an increased risk (up to 1%) of osteonecrosis of the bones of the oral cavity (ONJ), ascribed to osteomyelitis due to* Actinomyces* infection. This event occurs very rarely in patients (1:10,000 treated patients) receiving bisphosphonate or denosumab therapy at the regimens commonly employed in osteoporosis [142]. For subjects treated with bisphosphonates for osteoporosis for less than 3 years who do not have individual risk factors (diabetes, immunosuppression, steroids, smoking, alcohol), the risk of ONJ for invasive procedures is extremely low. In the case of surgery in the oral cavity (extraction), a broad-spectrum antibiotic therapy is mandatory in order to prevent bone infection (grade B recommendation). Many guidelines suggest the discontinuation of BPs for a period of 3 months and the recovery of the drug upon the healing of the surgical wound. There is no evidence that this actually reduces the risk of ONJ in view of persistent pharmacological effects of bisphosphonates. For the same reason, moreover, the suspension of bisphosphonate for a relatively short period of time (1/2 months) probably does not compromise the effectiveness of the therapy for osteoporosis. The Ministry of Health has recently produced a document concerning ONJ associated with the use of BPs on both oncological and osteoporotic patients under the auspices of the Society of Maxillofacial Surgery and Pathology and Oral Medicine (SICMF and SIPMO). It should be stressed that many of the recommendations derived from the literature and present in many international guidelines have a relatively low level of evidence but a relatively high recommendation based on expert consensus. All patients should be evaluated for ONJ risk factors prior to antiresorptive treatment, and a dental examination with appropriate preventive dentistry should be considered prior to treatment in patients with concomitant risk factors. Patients should be encouraged to maintain good oral hygiene practices, receive routine dental check-ups, and immediately report any oral symptoms such as dental mobility, pain, or swelling during treatment. While undergoing treatment, these patients should avoid invasive dental procedures if possible, but bisphosphonate or denosumab therapy should not be regarded as a contraindication for necessary dental treatment. In the vast majority of patients, the benefits of treatment outweigh the risks (grade A recommendation).

Atypical fractures, mainly of the subtrochanteric and diaphyseal regions of the femoral shaft, have been reported in patients on long-term therapy with bisphosphonates or denosumab on rare occasions. In patients treated with BPs for many years (as well as in patients with no previous exposure to bisphosphonates), the appearance of atypical (transverse) subtrochanteric femoral fractures was reported. The incidence of these fractures during long-term BP therapy is very low (3.2–50 cases per 100,000 person-years), but they are clearly linked to the duration of therapy. Based on the data available and due to the rarity of these events, the benefits of antiresorptive therapy outweigh the risk. In order to minimize the risk of subtrochanteric fracture in patients treated with bisphosphonates, the following may be useful: (a) consider periods of “therapeutic vacation” after careful consideration of the benefit–risk ratio, and (b) monitor and correct other risk factors for atypical fracture (chronic use of corticosteroids, hypovitaminosis D, chronic use of proton pump inhibitors, the presence of several skeletal diseases, osteoporosis) (grade B recommendation).

During bisphosphonate or denosumab therapy, patients should be advised to report any unexplained thigh, groin, or hip pain; if such symptoms develop, imaging of the femur (X-ray, isotope scanning, or MRI) should be performed. If an atypical fracture is present, the contralateral femur should also be imaged (grade A recommendation).

Discontinuation of bisphosphonate or denosumab therapy should be considered in patients who develop an atypical fracture, and alternative treatment options should be considered where appropriate. Surgical treatment with intramedullary nailing is often recommended.

#### Selective estrogen receptor modulators (SERMs)

SERMs are synthetic compounds that bind to the receptor for estrogen and produce agonistic effects in bone and liver but antagonistic effects at the level of the breast and genitourinary tract.

The SERMs that are currently approved in Italy for the prevention and treatment of osteoporosis are raloxifene and bazedoxifene [143]. They are contraindicated in women with childbearing potential, a history of venous thromboembolism or unexplained uterine bleeding, liver and kidney failure, or climacteric symptoms.

Raloxifene is a selective estrogen receptor modulator that inhibits bone resorption. It is approved for the treatment and prevention of osteoporosis in postmenopausal women at a dose of 60 mg daily. It has been shown to reduce vertebral fracture risk but reductions in nonvertebral and hip fractures have not been demonstrated.

Bazedoxifene is able to prevent loss of bone mass at a dose of 20 mg/day in normal and osteopenic women. In women with osteoporosis, the risk for vertebral fracture was significantly reduced (by 42%). Extending the study to 5 years demonstrated the persistence of the effect on vertebral fractures (a 32% risk reduction). A post hoc assessment in high-risk patients allowed the demonstration of a significant risk reduction for nonvertebral fractures for both 3 and 5 years. In addition, bazedoxifene showed a greater antiestrogenic effect in the uterus in the absence of significant side effects.

Conversely, estrogen therapy is no longer indicated for osteoporosis therapy or the prevention of osteoporosis.

#### Teriparatide

Teriparatide is the active fragment (recombinant human PTH 1–34) of parathyroid hormone. During the first 12 months of teriparatide therapy, it markedly stimulates bone formation; this period is therefore termed the “anabolic window” of teriparatide. It is administered as a subcutaneous injection at a dose of 20 μg/day, and the duration of treatment is limited to 24 months. The effect on trabecular BMD is significantly greater than that obtained with bisphosphonates, with an increase in vertebral BMD at 18 months of close to 10%. In addition, teriparatide induces an improvement in certain geometric features of cortical bone related to resistance to fracture. It is approved for the treatment of osteoporosis in postmenopausal women at a high risk of fracture and is given as a subcutaneous injection at a dose of 20 μg/day (grade A recommendation). Teriparatide is also approved for the treatment of osteoporosis associated with systemic glucocorticoid therapy in women with an increased risk of fracture. The duration of treatment is limited to 24 months. It has been shown to reduce the frequency of vertebral and nonvertebral fractures in postmenopausal women with osteoporosis, but there does not appear to be any data on hip fractures [144].

Teriparatide is contraindicated in patients with hypercalcemia, hyperparathyroidism, severe renal impairment, prior radiation to the skeleton, and malignant disease affecting the skeleton. It should be used with caution in patients with moderate renal impairment. Side effects include headache, nausea, dizziness, and postural hypotension.

#### Strontium ranelate

Strontium ranelate is a molecule that contains two atoms of strontium linked to ranelic acid. Treatment with strontium ranelate is effective at reducing the risk of vertebral, nonvertebral, and hip fractures in women with postmenopausal osteoporosis. Strontium ranelate has been evaluated in two clinical trials with durations of 5 years—with the main analysis carried out after 3 years—involving more than 7000 women. The results at 3 years showed that the drug reduced the risk of vertebral, nonvertebral, and hip fractures (in a high-risk subgroup) by 41, 16, and 36%, respectively. The results at 5 years confirmed the results observed in the first 3 years. The drug modestly increases bone formation markers (ca. 15%) while simultaneously reducing those of bone resorption (10–15%) [145, 146].

Treatment with strontium ranelate leads to a modest change in bowel habits and is associated with a slight increase in thromboembolic risk, particularly in elderly patients. The drug is contraindicated in patients with current or previous venous thromboembolism (VTE), and in patients who are temporarily or permanently immobilized. The need to continue treatment in patients who are > 80 years old and at risk of VTE should be reevaluated. Rarely, serious skin allergic reactions to this drug have been reported, sometimes associated with potentially fatal systemic symptoms (e.g., drug reaction with eosinophilia and systemic symptoms (DRESS); Stevens–Johnson syndrome; toxic epidermal necrolysis). In such cases, the drug must be immediately and permanently suspended (EMA/185,175/2012).

In a post hoc analysis, treatment with strontium ranelate was also associated with an increased risk of myocardial infarction in a subgroup of patients with an increased baseline cardiovascular risk (relative risk compared to placebo group: 1.6 (95% CI [1.07; 2.38]).

For the above reasons, the use of this drug is now restricted to severe osteoporosis in postmenopausal women and men at a high risk of fracture who cannot be treated with other approved drugs. It should not be used in patients with an established, current, or past history of ischemic heart disease, peripheral arterial disease, and/or cerebrovascular disease, or those with uncontrolled hypertension (grade A recommendation).

### Approved drugs for male osteoporosis and indication for treatment

Alendronate, risedronate, zoledronate, teriparatide, denosumab, and strontium ranelate are approved drugs for the treatment of male osteoporosis in Europe.

BPs are able to increase bone mass at the spine and the hip and reduce the risk of vertebral fracture in male idiopathic osteoporosis and glucocorticoid-induced osteoporosis. Risedronate is also indicated for the treatment of osteoporosis in men at a high risk of fracture. Zoledronic acid has also been demonstrated, for the first time, to reduce the risk of clinical fracture and mortality when given to patients shortly after their first hip fracture (grade A recommendation).

Denosumab is indicated for male idiopathic osteoporosis and iatrogenic osteoporosis due to androgen deprivation therapy in prostate cancer. Indeed, it is able to increase the BMD in males at a high risk of fracture and is indicated in the treatment of bone loss in subjects on androgen deprivation therapy for prostate cancer (grade A recommendation).

Teriparatide is indicated in severe osteoporosis or when new vertebral or hip fractures occur during treatment with other approved drugs for osteoporosis (grade A recommendation).

The safety profile of these drugs and their effects are comparable with the incidence and type of adverse events recorded in post-menopausal female population [147].

### Treatment of glucocorticoid-induced osteoporosis

Chronic exposure to excess exogenous or endogenous glucocorticoids is an important cause of osteoporosis and fractures. Glucocorticoids stimulate bone resorption and reduce bone formation by inhibiting the proliferation and differentiation of osteoblasts and promoting the apoptosis of osteoblasts and osteocytes. Moreover, they alter the calcium balance, reducing intestinal absorption, increasing renal excretion, and reducing the secretion of androgens and estrogens, especially pituitary gonadotropins. Bone loss caused by glucocorticoid treatment starts early (in the first few weeks) and is more pronounced in the first 6–12 months, especially at the trabecular bone (spine) level, with an increased risk of low-trauma fractures. Fragility fractures occur in 30–50% of patients within the first 5 years of chronic glucocorticoid therapy. The probability of fracture is further increased if additional risk factors are present in the same subject. The International Osteoporosis Foundation and the European Society of Calcified Tissues have published a framework for the development of national guidelines for the management of glucocorticoid-induced osteoporosis (GIO) in men and women aged 18 years and over in whom continuous oral glucocorticoid therapy is considered for 3 months or longer [148]. Alendronate, risedronate, zoledronic acid, and denosumab are approved for the prevention of fractures during chronic treatment with glucocorticoids or when chronic treatment with glucocorticoids lasting more than 3 months is planned (prednisone equivalent dose ≥ 5 mg/day) (grade A recommendation). Teriparatide is the optimal choice in patients with established major fragility fractures who are receiving long-term glucocorticoid treatment (grade A recommendation).

### Treatment of osteoporosis in patients with CKD and after transplantation

Patients with chronic kidney disease (CKD) who are undergoing hemodialysis show an incidence of hip fracture that is threefold higher than that for the general population. Vertebral fractures occur in 50% of subjects receiving periodic hemodialysis. Mortality in the first year after hip fracture doubles with respect to the general population.

In subjects with CKD stage 1–3, alendronate, risedronate, teriparatide, and denosumab prevent fragility fracture with the same degree of efficacy and safety as for subjects with normal renal function. Bisphosphonates and teriparatide have not been adequately investigated in subjects with CKD stages 4–5 and 5D, and are generally contraindicated in cases of stage IV CKD (creatinine clearance below 30 ml/min) (grade A recommendation). Denosumab can be administered even to patients with advanced renal failure. Preliminary studies have demonstrated its efficacy in a group of patients with CKD stage 4, but it has not been possible to draw any definitive recommendations so far (grade C recommendation). The progressive deterioration in renal function induces a significant decrease in the active metabolite of vitamin D, i.e., calcitriol, resulting in increased levels of parathyroid hormone. In subjects with CKD stages 4–5 and 5D with elevated parathyroid hormone, calcitriol and its analogs are able to reduce the levels of parathyroid hormone and favorably modify the alterations in bone metabolism (grade A recommendation). Treatment with cholecalciferol is able to consistently and significantly reduce the levels of parathyroid hormone in subjects with CKD stages 1–5 and 5D [149].

In organ transplantation, long-term immunosuppressive and glucocorticoid therapies that are usually commenced at high doses soon after the procedure greatly increase the risk of fragility fracture in these patients. Data show a prevalence of fragility fractures of 10–15% in patients waiting for an organ transplant (kidney, heart, liver, or lung), with an increase in prevalence after transplantation of up to 50%.

Alendronate, pamidronate, ibandronate, and zoledronic acid have been shown to increase bone mass in the absence of significant adverse events and, in particular, without inducing any alteration in renal function in patients with mild renal impairment after renal transplantation. Several studies in small cohorts of patients have shown that the administration of intravenous ibandronate, pamidronate, and zoledronic acid has a prophylactic effect regarding vertebral fractures in the absence of significant side effects, without incurring substantial variations in kidney function (grade C recommendation). Hypovitaminosis D is present in about 80% of patients with organ transplantation, and treatment with cholecalciferol and calcidiol is strongly recommended using schemes and dosages employed in the general population [150] (grade A recommendation).

### Treatment of juvenile osteoporosis

There is no officially approved treatment for patients with IJO. The effect of any kind of medical intervention is difficult to judge because the disease is rare, has a variable course, and is generally believed to resolve without treatment. Some papers report an increase in BMD and clinical improvement after treatment with bisphosphonates (grade C recommendation).

Bisphosphonate intervention should be restricted to children with multiple vertebral crush fractures, who may also experience debilitating chronic bone pain [151–153]. Medical therapies should complement orthopedic and rehabilitative measures such as physiotherapy in all such cases.

Neridronate is the only bisphosphonate registered for the treatment of osteogenesis imperfecta. It is also used in all forms of IJO, meaning that it is not necessary to resort to expensive and not easily accessible genetic evaluations (grade B recommendation).

### Policy for reimbursement in Italy

In Italy, the Ministry of Health and the Italian Drug Agency recently revised the criteria for the prescription and reimbursement of antiosteoporotic drugs [154]. Three main categories were identified:

- Secondary prevention in patients with a history of one or more previous fragility fractures at major sites (hip or spine) or at minor sites plus T-score < − 3
- Primary prevention in postmenopausal women and men over 50 years of age who are undertaking pharmacologic treatments that are detrimental to bone health (long-term glucocorticoids, antihormonal treatments for mammary and prostate cancer) or are at a high risk for fracture (T-score < − 4, or T-score < − 3 plus additional risk factors).

For secondary prevention, alendronate, risedronate, and zoledronate are the first-choice drugs, while denosumab and strontium ranelate are the second and third choices, respectively. In severe cases (≥ 3 major fractures, or ≥ 1 major fracture plus T-score < − 4, or ≥ 1 major fracture plus chronic glucocorticoids, or ≥ 1 major fracture under an approved antiosteoporotic treatment for more than 1 year), teriparatide is the first choice. Denosumab is indicated as a second-choice drug in the secondary prevention of refractures, and can be prescribed in the presence of contraindication or proven side effects of and further fractures under the approved first-choice treatment.

For primary prevention, alendronate, risedronate, and zoledronate are indicated as first-choice drugs for bone protection under chronic glucocorticoid treatment, while risedronate or alendronate represent the first-choice drugs in patients with T-scores of less than − 4 or less than − 3 plus a high-risk factor (in this latter case, zoledronate, ibandronate, raloxifene, and bazedoxifene are indicated as second-choice drugs, and strontium ranelate as the third choice).

In addition to alendronate, risedronate, zoledronate, and denosumab are the first-choice drugs for patients with breast cancer or prostate cancer receiving adjuvant hormonal blockade.

A subsequent change from the first-choice treatment could be made in the presence of intolerance, an inability to achieve the required intake of the drug, side effects of or contraindications for the first-choice drug, or, in the case of teriparatide, if the end of the maximum allowable treatment period has been reached.

**Fig. 6:**
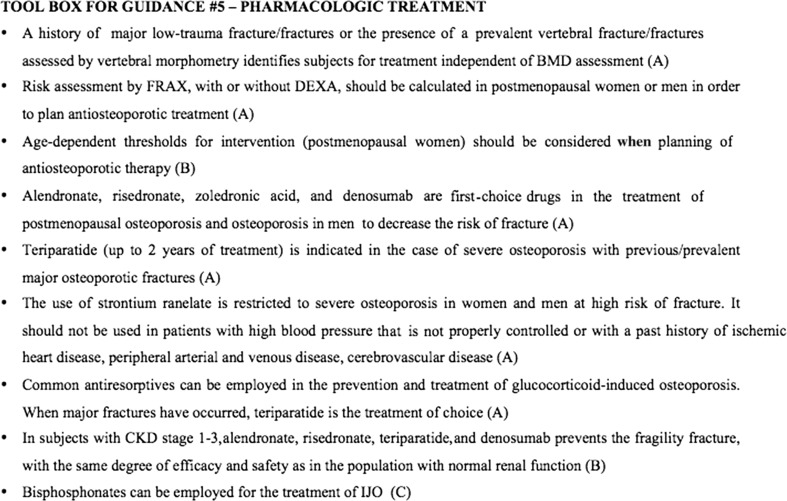
Toolbox for guidance: pharmacologic treatment

Figure 6 summarizes the recommendation statements for the pharmacologic treatment of osteoporosis in a toolbox.

## Integrated approaches for secondary prevention

Integrated and multidisciplinary approaches for the secondary prevention of refracture are strongly advised and are needed at all levels of assistance, such as in primary, secondary, and tertiary care settings. Connections between and within these different levels of care must be ensured to optimize the pathways of assistance for osteoporotic patients at a high risk of (re)fracture.

### Fracture liaison service

**Fig. 7:**
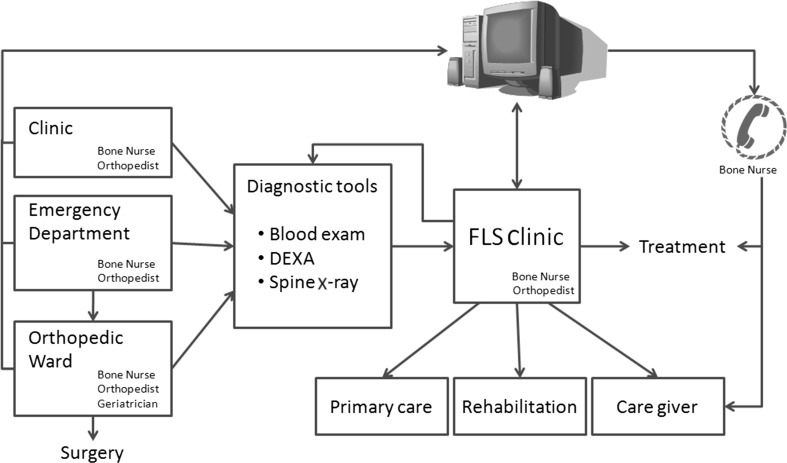
Model of a fracture liaison service (FLS)

The effectiveness of the diagnostic and therapeutic pathway described previously increases if it is incorporated within a structured program of tertiary prevention. Fracture liaison services (FLSs) are systems that are implemented by health care systems to prevent secondary fractures in osteoporotic patients (Fig. 7). FLSs were proposed in the framework of a project called Capture the Fracture, promoted by the Fracture Working Group of the Committee of Scientific Advisors of the International Osteoporosis Foundation (IOF) in 2011 [155]. They target patients with a fragility fracture because approximately 80% of those patients do not undergo screening for osteoporosis and are not treated with antiosteoporotic medications. Moreover, the aim of a FLS is to improve communication between primary care and medical specialists and facilitate the approval of care pathways for osteoporosis and treatment for fragility fractures. Primary care physicians, orthopedic teams, and other specialized physicians with expertise in fragility fracture prevention are coordinated in a FLS. Such a service also includes a dedicated caseworker and a clinical nurse specialist who follows the treatment of patients with a fragility fracture. FLSs are based in primary or secondary health care settings. Different models of care have been planned, with the aim of establishing an effective method of obtaining recommended standards of treatment for osteoporotic fractures.

Evidence suggests that two-thirds of all services that are designed to prevent secondary fracture utilize a chief who acts as a link between the patient and the health care system [156], as this improves the communication between health-care figures, providing that the patient follows a well-defined care pathway.

Scientific evidence underlines that the FLS concept is an established and proven method of preventing secondary fragility fractures, following osteoporosis treatment, and reducing the overall costs of fracture treatment. Miller et al. [156] noted that the strength of the FLS concept is that it facilitates patient care by automatically directing subjects with a fragility fracture to a healthcare system that is able to provide them with the intervention they need and that helps them to prevent avoidable fracture-related complications or readmission to hospital. Using this approach, patients can be treated in greater numbers and those treated show increased treatment adherence to treatment, a decreased risk of secondary fracture, and even reduced mortality over time. The core of the FLS program is based on a physician, a FLS coordinator, and a nurse. The orthopedic surgeon is also located at the core of the FLS, since they are often the first to take care of a patient with a fragility fracture and his family, and they are the first to explain the link between the fracture and osteoporosis at the time of admission to the hospital. The orthopedic surgeon may provide the bridge between the patient and the bone specialist, enabling the initiation of effective programs of secondary prevention of refracture. Patients usually do not consult their primary care physician after their fracture has healed, and they often do not require further interventions for bone fragility nor antiosteoporotic treatment. Aizer et al. [157] reviewed factors contributing to the treatment gap in osteoporosis, and suggested that effective bone health management after a fracture necessitates a multimodal approach and requires the recognition of a high risk of fracture, effective communication with patients about the identification of risk factors for fragility fracture, and the importance of patient adherence to treatment regimens. Fragility fracture management starts with the identification of patients diagnosed with a fragility fracture through bone health consultations. Their bone status must be evaluated based on their medical history, physical examination, and laboratory exams. Recommendations regarding the treatment for osteoporosis—if necessary—are supplied to the patient, including calcium and vitamin D supplementation and lifestyle modifications. The fragmentation of the care path contributes to failure; this is highlighted by the discrepancy between the providers involved in hospitalization and those involved in subsequent care management. It was demonstrated that only 40% of women over 60 years of age with a fragility hip fracture reported an awareness of a diagnosis of osteoporosis at their hospital discharge [158]. A good FLS model should be based on both a diagnosis of osteoporosis made by the orthopedic surgeon at the time of the admission for the trauma and the effective communication of that diagnosis to the patient. This should always be followed by recommendations regarding individual fracture risk modifications, including pharmacologic and nonpharmacologic approaches and physical therapy. The latter step is represented by a bone health follow-up, ensuring clear communication with primary care providers regarding the assessment and recommendations for bone fragility treatment. Mitchell et al. [159] remarked how the FLS model has been shown to be able to eliminate the care gap in a clinical and cost-effective manner. Indeed, the increase in secondary fracture prevention methods leads to a rational stepwise pathway to improving health gains. The keywords for long-term preventive care are recognition, examination, and initiation (of intervention):

- Recognition: the correct recognition of a fragility fracture when a patient is admitted to the hospital
- Examination: the evaluation of bone mineral density by DEXA examination, and dorsal and lumbar spine X-rays for nonvertebral fractures
- Initiation: pharmacologic treatment of osteoporosis, in addition to nonpharmacological therapy and falls prevention.

The crucial point is that adherence to osteoporosis treatment has been shown to decrease rapidly in around half of the patients who start the treatment, and there is a lack of clarity regarding the clinical responsibility for osteoporosis treatment [160]. This is because orthopedic surgeons take care of the acute phase of the fracture and do not usually treat the underlying disease, while primary care physicians do not investigate patients who have recently suffered fragility fractures unless there is a specific recommendation to do so by a hospital specialist. Cost-effectiveness analysis of a well-organized FLS indicates that there is a large decrease in the incidence of secondary hip fracture in the first year. Clinical trials confirm the effectiveness of FLS. In particular, they document a 30% reduction in second fractures and a 40% reduction in major fractures (hip, humerus, spine, and pelvis) based on a 3-year follow-up in FLS units [161]. In addition, a FLS that integrates different specialists can provide early diagnosis of psychiatric disease, better psychiatric care, and earlier discharge, with a reduction in hospital costs and hospitalization length [162]. Compared with pure primary care, FLS leads to better compliance with osteoporosis treatment. In fact, it seems that it is useful to apply the FLS approach when starting and maintaining therapy for osteoporosis [163]. In particular, a minimal trauma fracture liaison (MTFL) service significantly reduces the risk of refracture by 80%, leading to very high cost-effectiveness [164]. Multicenter studies have evaluated patients with a recent fragility fracture. Their results have shown that 88% of the patients enrolled in a FLS in four Dutch hospitals were complying with their osteoporosis treatment at the 1-year follow-up, and only 2% of the patients had a subsequent fragility fracture [165]. In the UK, the presence of a FLS leads to a high percentage of patients being diagnosed and treated for osteoporosis after a hip or proximal humeral fracture. The study revealed that 85% of patients with a proximal humeral fracture and 20% with a hip fracture underwent a DEXA scan [166]. A survey of five large FLSs in the Netherlands highlighted some critical aspects, such as differences in the selection of patients and the evaluation of clinical risk factors.

In conclusion, evidence suggests that a FLS is useful for achieving optimal osteoporosis management and preventing secondary fragility fractures, but it needs to be well organized, and patients should be enrolled in the program when they are first admitted to the hospital to treat a fragility fracture (grade A recommendation).

### The role of the bone care nurse

The bone care nurse (BCN) is a nurse who has acquired advanced knowledge of metabolic bone diseases and specific clinical skills to evaluate, plan, and manage people affected by osteoporosis [167]. These skills allow the BCN to implement the optimal paths for the education, diagnosis, treatment, and rehabilitation of osteoporotic patients. BCNs work in various care settings in hospitals and communities, such as in the areas of prevention, primary care, and rehabilitation. BCNs organize and participate in educational campaigns in order to encourage a culture of specific prevention through early diagnosis, the appropriate use of diagnostic tools to ensure long-term adherence to a proper lifestyle, and the proper use of drug therapy. In addition, BCNs with multidisciplinary teams plan and manage clinical care pathways (FLSs) and monitor people at risk for osteoporosis or fragility fracture through tailored educational intervention [168]. In fact, this educational intervention is considered key to improving adherence in osteoporotic patients. The relevant literature shows that, during follow-up, tailored educational intervention along with counseling, motivational interviews, and educational programs are more effective than standard information at improving the outcomes of osteoporotic patients [169]. These tailored educational interventions are performed to promote a healthy lifestyle and improve adherence to an appropriate diet, regular exercise, and the proper use of drug therapy. Nurses should encourage patients to stop smoking, ensure their diet includes the appropriate nutrition, avoid a sedentary lifestyle, take regular medication, and spend at least 10–15 min outdoors on sunny days. These are all behaviors that will help the patient to maintain a healthy lifestyle and improve self-care.

Several studies have shown that decision aids, monitoring schedules with nurses, and pharmaceutical care with counseling packages are interventions that improve adherence to drug therapy [170–172]. Specifically, three studies used interventions involving direct relationships between patients and health professionals, such as counseling and telephone counseling interventions, to develop therapeutic relationships [173, 174]. In another study, adherence to medication was improved when tailored educational intervention led by a nurse was included [175]. Four studies showed that medication adherence did not improve with delivery to the patient of educational materials such as leaflets, letters, or automated telephone calls [176–178]. Three studies that focused on the promotion of adequate nutrition and exercise levels showed that it is possible to encourage healthy lifestyles through therapeutic lifestyle modification intervention, a tailored intervention with written materials, counseling sessions, and an exercise education program based on the transtheoretical change model [179–181]. According to the scientific literature, switching to an appropriate diet with the proper calcium and vitamin D intake and ensuring that a sufficient amount of physical activity is performed are important lifestyle changes that can decrease the risk of fracture [182–184]. Moreover, the literature shows that interventions which focus only on information do not decrease risk factors for falls and do not improve adherence to healthy lifestyles [185]. In this context, BCNs could play a key role in the lives of patients affected by osteoporosis or those who have suffered a fragility fracture (grade B recommendation).

### Relationship with the primary care system

Despite numerous campaigns promoted by various scientific societies, primary care physicians (PCPs) do not yet perceive osteoporosis to be a serious disease in the elderly. Previous studies [164, 166] have shown that, if not directly prescribed at discharge, many patients with a fragility fracture do not receive a prescription for diagnostic tests from their primary care physician, and antiosteoporotic therapy is often not prescribed. The PCP is a fundamental figure in fracture risk management. Knowledge of the patient’s comorbidities can allow the doctor to identify bone fragility even before a fracture occurs.

PCPs recognize and treat osteoporosis rather infrequently, for a number of reasons. Usually, osteoporosis is not considered a particularly important disease in the elderly, or important enough that it needs to be addressed in a specific visit. Furthermore, physicians have the opportunity to record medications but they may not consider supplements that do not need a prescription, such as calcium and vitamin D. Osteoporosis is an asymptomatic disease that is difficult to diagnose in the absence of fracture. When fractures occur, patients are often treated for the fracture, but only rarely is the trauma mechanism investigated. If the orthopedic surgeon and the PCP both fail to identify a fracture as a fragility fracture, the bone metabolism is not studied and so osteoporosis is not diagnosed. Even in cases of evident spinal deformity, patients are often not treated until vertebral fractures do not produce symptoms such as chronic pain. Dual energy X-ray absorptiometry (DEXA) is the gold standard for the diagnosis of osteoporosis, but this exam is not always prescribed, even after a fragility fracture or in the presence of risk factors. Also, even when a diagnosis of osteoporosis has been made, physicians do not always prescribe drugs to reduce the risk of future fractures. This situation is responsible for more than 240,000 hospital admissions in Italy each year, which costs the health system €1.5 billion. For this reason, osteoporosis should be considered a public health priority.

In conclusion, after a fragility fracture, the PCP should be involved in a tertiary prevention program (FLS). In fact, as they have had the opportunity to follow the patient in detail, outside of the hospital, and given their knowledge of the patient’s overall clinical course, the PCP has the opportunity to check that the patient is correctly adhering to the prescribed therapy and to monitor for the onset of more severe conditions, thus acting as a natural link between the patient, the orthopedist, and the bone specialist (grade A recommendation).

**Fig. 8:**
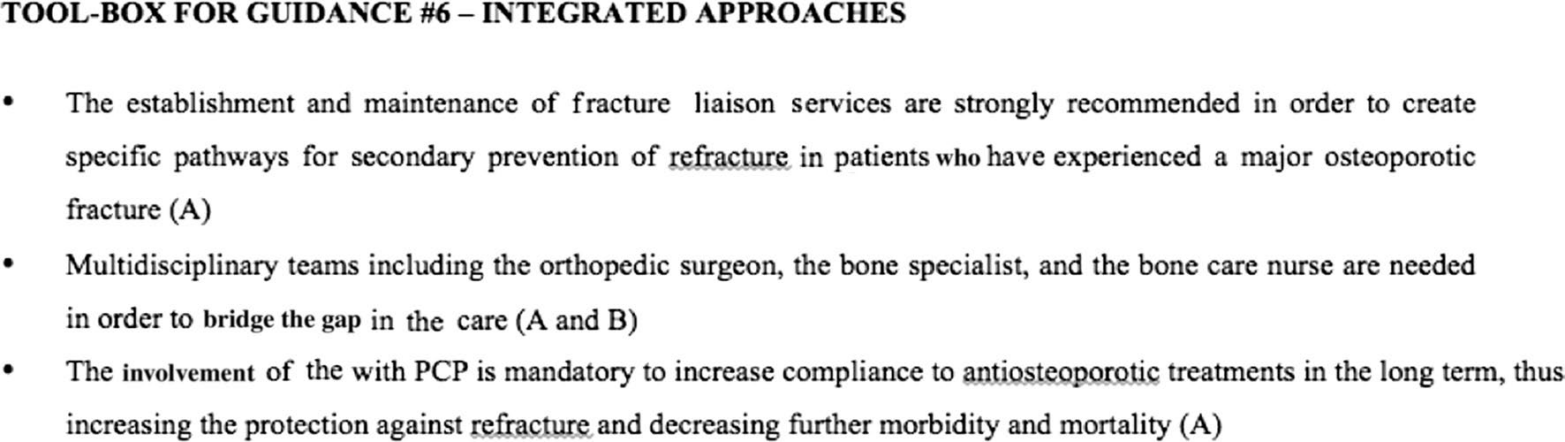
Toolbox for guidance: integrated approaches

Figure 8 summarizes the recommendation statements for integrated approaches to osteoporotic fractures as a toolbox.

## Conclusions

The Italian Society for Orthopaedics and Traumatology has produced up-to-date Italian guidelines for the primary, secondary, and tertiary prevention of osteoporosis and osteoporotic fractures. This guidance is primarily addressed to Italian orthopedic surgeons, but should also prove useful to other bone specialists and general practitioners who wish to optimize the diagnosis, prevention, and treatment of osteoporosis and its consequences. More effective interactions between the various health professionals involved in this field are needed to improve outcomes, as demonstrated by the successful implementation of FLS throughout Europe. Comprehensive assessment of the risk of fracture, beyond simply assessing the BMD, is crucial to any program aiming at the primary, secondary, and tertiary prevention of osteoporosis.
